# Association and progression of multi-morbidity with Chronic Kidney Disease stage 3a secondary to Type 2 Diabetes Mellitus, grouped by albuminuria status in the multi-ethnic population of Northwest London: A real-world study

**DOI:** 10.1371/journal.pone.0289838

**Published:** 2023-08-25

**Authors:** Rakesh Dattani, Zia Ul-Haq, Moulesh Shah, Gabrielle Goldet, Lord Ara Darzi, Hutan Ashrafian, Tahereh Kamalati, Andrew H. Frankel, Frederick W.K. Tam

**Affiliations:** 1 Imperial College London, London, United Kingdom; 2 Imperial College Health Partners, London, United Kingdom; 3 Imperial College Healthcare NHS Trust, London, United Kingdom; Karolinska Institutet, SWEDEN

## Abstract

**Introduction:**

The prevalence of Diabetic Kidney Disease (DKD) secondary to Type 2 Diabetes Mellitus (T2DM) is rising worldwide. However, real-world data linking glomerular function and albuminuria to the degree of multi-morbidity is lacking. We thus utilised the Discover dataset, to determine this association.

**Method:**

Patients with T2DM diagnosed prior to 1^st^ January 2015 with no available biochemical evidence of CKD were included. Patients subsequently diagnosed and coded for CKD3a in 2015, were grouped by the degree of albuminuria. Baseline and 5-year co-morbidity was determined, as were prescribing practices with regards to prognostically beneficial medication.

**Results:**

We identified 56,261 patients with T2DM, of which 1082 had CKD stage 3a diagnosed in 2015 (224-CKD3aA1,154-CKD3aA2,93-CKD3aA1; 611 patients with CKD3a but no uACR available in 2015 were excluded from follow up). No statistically significant difference was observed in the degree of co-morbidities at baseline. A significant difference in the degree of hypertension, retinopathy, ischaemic heart disease and vascular disease from baseline compared to study end point was observed for all 3 study groups. Comparing co-morbidities developed at study end point, highlighted a statistical difference between CKD3aA1 Vs CKD3aA3 for retinopathy alone and for hypertension and heart failure between CKD3aA2 Vs CKD3aA3. 40.8% of patients with CKD3aA2 or A3 were prescribed Renin Angiotensin Aldosterone inhibitors (RAASi) therapy between June-December 2021. Survival analysis showed 15% of patients with CKD3aA3 developed CKD stage 5 within 5 years of diagnosis.

**Discussion:**

CKD3a secondary to DKD is associated with significant multimorbidity at baseline and 5 years post diagnosis, with CKD3aA3 most strongly associated with CKD progression to CKD 5, heart failure, hypertension and retinopathy compared to CKD3aA1 or CKD3aA2 at 5 years post diagnosis. The lack of uACR testing upon diagnosis and poor prescribing of RAASi, in those with CKD3aA2/A3, raises significant cause for concern.

**Conclusion:**

DKD is associated with significant multimorbidity. Significant work is needed to be done to ensure patients undergo testing for uACR, to allow for future risk stratification and ability to be started on prognostically beneficial medication.

## Introduction

Chronic Kidney Disease (CKD) is defined by the presence of 1 or both of a reduced glomerular filtration rate (GFR) and an elevated urine albumin creatinine ratio (uACR) for at least a period of three months [1]. Whilst an array of causes exist, Type 2 Diabetes Mellitus (T2DM) accounts for approximately 50% of all CKD, with CKD itself affecting 15% of the worldwide population [2]. Alongside being associated with CKD, patients with T2DM are known to be at risk of an array of other micro- and macro-vascular complications, including extensive cardiovascular disease (CVD). With the prevalence of T2DM expected to reach 700 million people worldwide by 2045, a similar rise in micro-/macro-vascular complications including CKD is to be expected. Whilst the link between albuminuria and progressive CKD and CVD are well-recognised in specialised clinics, the linkage between varying degrees of albuminuria and the array of micro- and macro-vascular complications of diabetes is less well established in the real-world setting [3]. Whilst only 10% of those that develop DKD are expected to reach end stage kidney disease (ESKD) requiring renal replacement therapy or palliation, 90% of patients with DKD are expected to die prematurely on account of CVD [2, 4, 5]. An enhanced understanding of the association between albuminuria and morbidity would thus encourage regular screening for DKD and allow for intervention aimed at delaying progression to ESKD and preventing the premature morbidity and mortality related to CVD, to be implemented [6].

Annual screening of patients with T2DM for DKD, by performing a blood test to determine estimated Glomerular Function (eGFR) and a urine test to determine the uACR is recommended from the time of diagnosis with T2DM (as opposed to 5 years post diagnosis with Type 1 Diabetes Mellitus) [7, 8]. However, despite its recommendations, the National CKD audit showed only 54% of patients with diabetes had an annual uACR performed in primary care [9]. This lack of uACR testing would prevent the initiation of prognostically beneficial medication such as Renin Angiotensin Aldosterone Receptor inhibitors (RAASi), Sodium Glucose Cotransporter 2 inhibitors (SGLT2i) as well as the recently approved non-steroidal mineralocorticoid receptor antagonist Finerenone, all of which require a set threshold of albuminuria to be met prior to their initiation amongst patients with CKD [8]. This lack of assessment of albuminuria amongst patients with diabetes, has been proposed at least in part to be due to a lack of clarity amongst clinicians with regards to the association of varying degrees of albuminuria and the development of T2DM complications, in addition to the limited capacity of primary care to undertake uACR testing on an annual basis in all diabetics. With the rising prevalence of T2DM, ensuring primary care are better supported to fully screen patients for CKD by performing annual blood and urine testing, should be a key public health aim for both specialist renal clinicians and policy makers.

Given the lack of readily available data assessing the association between albuminuria and multimorbidity in T2DM, we employed the Discover dataset to interrogate real world data from the ethnically diverse population of Northwest London (NWL). The key aim of this study is to determine if there is a statistical difference in the development of co-morbidities of interest over the 5 year study period between patients with varying degrees of albuminuria with an eGFR between 45-59mls/min/1.73m^2^ (CKD3a) whilst providing real-world data on the prevalence of micro-/macro-vascular complications of T2DM and the prescription of prognostically beneficial medication in NWL, an area of vast ethnic and social diversity.

## Research design and methods

### Data source–Discover dataset

The data source utilised in this longitudinal retrospective cohort study is the Discover dataset. The Discover dataset is coded primary care, secondary, acute, mental health, community health and social care records for over 2.3 million patients who live and are registered with a General Practitioner (GP) in NWL. It is de-identified to meet data minimisation standards of the Information Standards Boards of NHS Digital and has an established governance structure. Whilst smaller in size when compared to the Clinical Practice Research Datalink (CPRD), it is more ethnically diverse whilst being comparable in terms of overall age, gender distribution and chronic disease prevalence in the United Kingdom [10]. The Discover dataset is accessible via Discover-NOW Health Data Research Hub for Real World Evidence through their data scientist specialists and Information Governance committee-approved analysts, hosted by Imperial College Health Partners. Discover-NOW have secured Health Research Authority (HRA) approval, with researchers not required to seek further ethical approval for use of the Discover Research Platform for research purposes for studies submitted to and approved by the NWL Data Access Committee. (REC reference ‐ 18/WM/0323; IRAS project ID ‐ 253449.

### Patient and public involvement

Due to the nature of this study, no patient or public involvement was undertaken.

#### Study subjects and design

In this study, the Discover dataset was retrospectively analysed between January 2015 and December 2021. The dataset was accessed and analysed by data analysts (Z.U.H and T.K.). Data was collected to understand the potential associations between CKD stage 3a with various stages of albuminuria and the development of other co-morbidities associated with T2DM and CKD. Disease states were identified based on patients being coded in primary or secondary care using ICD-10 or Read Codes version 2 (S2 File).

Patients aged over 40 years of age, coded for T2DM on their electronic health records prior to the 1^st^ of January 2015, for whom there were computerised records within the Discover dataset from 1^st^ of January 2015 were identified. Patients with an eGFR <60mls/min/1.73m^2^ or a known diagnosis of CKD stage 1 to 5 as defined by Kidney Disease Improving Global Outcomes (KDIGO), prior to 1^st^ January 2015 were excluded as were patients diagnosed with T2DM younger than 40 years of age to ensure accuracy of T2DM diagnosis and potential inaccurate recording of other forms of diabetes as T2DM. Patients with a new diagnosis of CKD3a signifying an eGFR between 45-59mls/min/1.73m^2^ between January to December 2015 were grouped by the degree of albuminuria (CKD3aA1 i.e. uACR <3mg/mmol, CKD 3aA2 i.e., uACR between 3-30mg/mmol and CKD 3aA3 ie. uACR >30mg/mmol). Patients without an uACR performed during the study entry year were excluded from longitudinal follow up.

For each patient, key demographic data including age, gender, ethnicity, geolocation, smoking, BMI, and deprivation score were collated. Baseline presence of micro- and macro-vascular complications of T2DM were collated as were the development of co-morbidities between 1^st^ January 2016 to 31^st^ December 2021. Pearson’s Chi-squared test with Yates’ continuity correction was used to determine the difference in the development of the co-morbidities of interest over the 5-year study period by the degree of albuminuria.

To determine practices with regards to prognostically beneficial medication prior to and at the end of the study period, prescription of RAASi (namely angiotensin converting enzyme inhibitors and angiotensin receptor blockers) for a minimum of 3 months in the 12 months prior to start of the study period (1^st^ January to 31^st^ December 2014), and the proportion of patients with a prescription for SGLT2i or RAASi or both in the last six months of the study period (1^st^ June to 31^st^ December 2021) were captured.

## Results

### T2DM patient characteristics

We identified 56,261 (25,045 Females Vs 31,216 Males) unique patients within the dataset, with a diagnosis of T2DM prior to 1^st^ January 2015. Amongst these, 41.58% of patients were diagnosed with T2DM in the five years prior to 2015, with the age in 2015 equally distributed between 40 and 90 years of age. The ethnicity of patients was representative of the local population of NWL, with Asian/Asian British (47%), White (32.4%) and Black/black British (11.9%) being the 3 most prominent ethnic groups with T2DM. Smoking status was poorly represented with only 15,607 patients (27.7%) with a documented smoking status (6,750 –smoker Vs 8857 –Ex-Smoker). Indices of multiple deprivation as a measure of social deprivation were consistent with the 2019 census for NWL with 67.2% of patients scoring between 3 and 7 (S1A-1C Table).

### Study cohort patient characteristics

Amongst the 56,261 identified patients with T2DM, 1,264 patients were identified to have with newly diagnosed CKD stage 3 (eGFR 30-59ml/min/1.73m^2^), between 1^st^ January and 31^st^ December 2015, of which 1082 had CKD stage 3a (eGFR 45-59ml/min/1.73m^2^) and 182 had CKD stage 3b (eGFR 30–44 ml/min/1.73m^2^). Patients with CKD3a were stratified according to degree of albuminuria (uACR <3 mg/mmol = normoalbuminuria, CKD3aA1; 3–30 mg/mmol = microalbuminuria, CKD3aA2; >30 mg/mmol = macroalbuminuria, CKD3aA3). 224 patients were identified to have CKD3aA1, 154 CKD3aA2 and 93 CKD3aA3. Amongst patients with CKD3a, 611 of 1082 (56.5%), did not have a uACR available between January-December 2015, and were excluded from longitudinal follow up. For both CKD3aA1 and CKD3aA2 approximately 40% were female and 60% male. CKD3aA3 were 30% female and 70% male. Patients with CKD3aA3 were more commonly of Asian or Asian British ethnicity than any other ethnicity, as opposed to CKD3aA1 and CKD3aA2 cohorts in whom 70–80% of cohort were either Asian/Asian British or of white ethnicity, representative of the local NWL population. (Table 1A and 1B).

**Table 1:** a. Age distribution of patients with newly coded CKD3a between 1/1/2015-31/12/2015, separated by the degree of albuminuria. CKD3aA1 = eGFR between 45-59mls/min/1.73m^2^ with uACR <3mg/mmol, CKD3aA2 = eGFR between 45-59mls/min/1.73m^2^ with uACR between 3-30mg/mmol and CKD3aA3 = eGFR between 45-59mls/min/1.73m^2^ with uACR >30mg/mmol. b. Ethnicity of patients with newly coded CKD3a between 1/1/2015-31/12/2015, separated by the degree of albuminuria. CKD3aA1 = eGFR between 45-59mls/min/1.73m^2^ with uACR <3mg/mmol, CKD3aA2 = eGFR between 45-59mls/min/1.73m^2^ with uACR between 3-30mg/mmol and CKD3aA3 = eGFR between 45-59mls/min/1.73m^2^ with uACR >30mg/mmol.

**a**								
	**Group 1**	**Group 2**		**Group 3**				
					
					
**Age in 2015 ‐ Years**	**CKD 3aA1**	**CKD 3aA2**		**CKD 3aA3**	**No uACR**		**Grand Total**	
40–44	<5	<5		<5	7		<11	
45–49	6	<5		10	15		<32	
50–54	14	10		12	51		87	
55–59	22	19		10	57		108	
60–64	22	12		14	70		118	
65–69	50	28		15	115		208	
70–74	37	33		13	103		186	
75–79	40	22		12	90		164	
80–84	18	17		<5	77		115	
85–89	11	8		<5	24		<44	
90+	<5	<5		<5	<5		<9	
**Grand Total**	**<224**	**<154**		**<93**	**<611**		**1,082**	
**b**								
	**CKD3aA1**			**CKD3aA2**			**CKD3aA3**	
**Ethnicity**	**Female**		**Male**	**Female**		**Male**	**Female**	**Male**
White	30		60	35		65	10	37
Asian or Asian British	45		39	15		52	11	9
Black or black British	15		22	<5		<19	<5	8
Other ethnic groups	<5		6	7		14	<5	7
Mixed	<5		<5	<5		<5	<5	<5
Null	-		-	-		<5	-	-
**Grand Total**	**<96**		**<128**	**<62**		**<92**	**<28**	**<65**

CKD3aA1 = eGFR between 45-59mls/min/1.73m2 with uACR <3mg/mmol.

CKD3aA2 = eGFR between 45-59mls/min/1.73m2 with uACR between 3-30mg/mmol.

CKD3aA3 = eGFR between 45-59mls/min/1.73m2 with uACR >30mg/mmol.

### Study cohort baseline co-morbidity

Hypertension, ischaemic heart disease (IHD), and diabetic retinopathy were overall the three most common co-morbidities noted in patients with T2DM and newly diagnosed CKD3a in 2015 (Table 2). Heart failure was more frequent in patients with CKD3aA3, than the other two groups of patients, but did not reach statistical significance when comparing CKD3aA1/CKD3aA2 Vs CKD3A3 at baseline (p = 0.26 and p = 1 respectively).The remainder of the clinical co-morbidities searched for (vascular disease, neuropathy, diabetic foot, cataract, liver disease, cerebrovascular disease, and amputation) were overall less prevalent at time of diagnosis with CKD3aA1/A2/A3.

**Table 2:** Comorbidities at baseline for each of the 3 study cohorts: CKD3aA1, CKD3aA2, and CKD3aA3. CKD3aA1 = eGFR between 45-59mls/min/1.73m^2^ with uACR <3mg/mmol, CKD3aA2 = eGFR between 45-59mls/min/1.73m^2^ with uACR between 3-30mg/mmol and CKD3aA3 = eGFR between 45-59mls/min/1.73m^2^ with uACR >30mg/mmol.

Co-morbidity	CKD staging		
	3aA1 ‐ 2015 n = 224	3aA2 ‐ 2015 n = 154	3aA3 ‐ 2015 n = 93
Hypertension	22%	29%	34%
Ischaemic heart disease	17%	18%	12%
Diabetic retinopathy	12%	19%	20%
Heart failure	5%	6%	14%
Vascular disease	3%	5%	4%
Neuropathy	2%	0%	0%
Diabetic foot	2%	1%	0%
Cataract	2%	5%	2%
Liver disease	1%	1%	0%
Cerebrovascular diseases ‐ Ischaemic	1%	1%	0%
Cerebrovascular diseases ‐ unspecified	0%	1%	0%
Amputation	0%	0%	0%
Cerebrovascular diseases ‐ haemorrhagic	0%	1%	0%

CKD3aA1 = eGFR between 45-59mls/min/1.73m2 with uACR <3mg/mmol.

CKD3aA2 = eGFR between 45-59mls/min/1.73m2 with uACR between 3-30mg/mmol.

CKD3aA3 = eGFR between 45-59mls/min/1.73m2 with uACR >30mg/mmol.

### Comparison of co-morbidity at study baseline

There was no statistical difference in the five commonest co-morbidities of interest (hypertension, diabetic retinopathy, IHD, heart failure and vascular disease) at baseline between CKD3aA1 Vs CKD3aA2, CKD3aA1 Vs CKD3aA3 and CKD3aA2 Vs CKD3aA3, highlighting the significant degree of retinopathy and CVD associated with CKD3a at the time of diagnosis, regardless of the degree of albuminuria (Table 3).

**Table 3:** Pearson’s Chi-squared test with Yates’ continuity correction, *p* values comparing co-morbidity occurrence according to degree of CKD at study baseline and end point within and between study groups. Statistical significance = *p* <0.05. CKD3aA1 = eGFR between 45-59mls/min/1.73m^2^ with uACR <3mg/mmol, CKD3aA2 = eGFR between 45-59mls/min/1.73m^2^ with uACR between 3-30mg/mmol and CKD3aA3 = eGFR between 45-59mls/min/1.73m^2^ with uACR >30mg/mmol.

Comorbidity	CKD3aA1 Vs 3aA2 Baseline	CKD3aA1 Vs 3aA3 Baseline	CKD3aA2 Vs 3aA3 Baseline	CKD 3aA1 Baseline vs CKD3aA1 Endpoint	CKD 3aA2 Baseline vs CKD 3aA2 Endpoint	CKD 3aA3 Baseline vs CKD 3aA3 Endpoint	CKD 3aA1 Endpoint vs CKD3aA2 Endpoint	CKD 3aA1 Endpoint vs CKD 3aA3 Endpoint	CKD 3aA2 Endpoint vs CKD 3aA3 Endpoint
**Hypertension**	0.3508	1	0.3805	<0.001	<0.001	<0.001	1	0.8817	*0*.*0453*
**Diabetic retinopathy**	0.7261	0.4015	1	<0.001	<0.001	0.001955	0.8815	*0*.*03944*	0.1337
**Ischaemic heart disease**	0.822	1	0.3185	<0.001	<0.001	0.001508	0.5498	0.4619	0.909
**Heart Failure**	0.2589	1	0.5096	<0.001	0.3738	<0.001	0.3626	0.3626	*<0*.*001*
**Vascular Disease**	1	1	1	<0.001	<0.001	<0.001	0.7402	0.8952	0.3112

Statistical significance = p<0.05.

CKD3aA1 = eGFR between 45-59mls/min/1.73m2 with uACR <3mg/mmol.

CKD3aA2 = eGFR between 45-59mls/min/1.73m2 with uACR between 3-30mg/mmol.

CKD3aA3 = eGFR between 45-59mls/min/1.73m2 with uACR >30mg/mmol.

### Longitudinal follow up from 2016–2021

**Fig 1:**
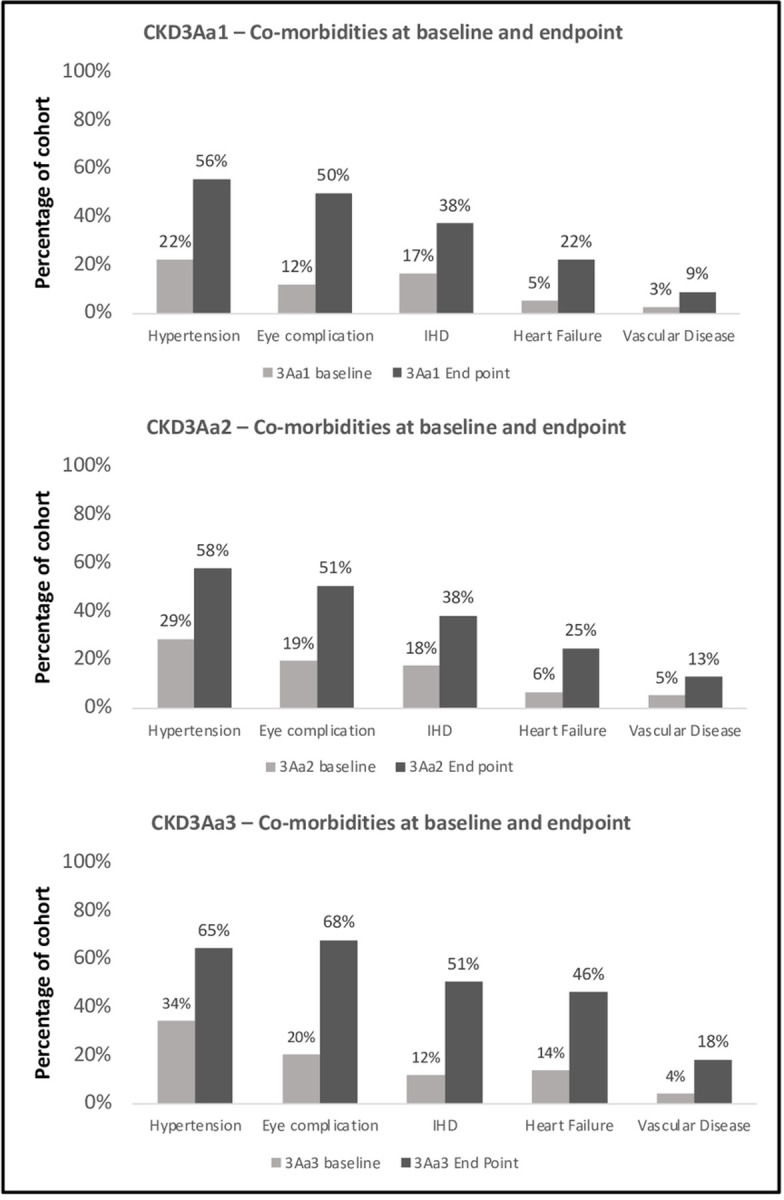
Co-morbidities at baseline and endpoint for each of the 3 study cohorts: CKD3aA1, CKD3aA2, and CKD3aA3. CKD3aA1 = eGFR between 45-59mls/min/1.73m^2^ with uACR <3mg/mmol, CKD3aA2 = eGFR between 45-59mls/min/1.73m^2^ with uACR between 3-30mg/mmol and CKD3aA3 = eGFR between 45-59mls/min/1.73m^2^ with uACR >30mg/mmol.

Patients with CKD3a -A1, A2, and A3 all had a high burden of disease, with all patients regardless of the degree of albuminuria having at least one of the co-morbidities of interest. Hypertension, diabetic retinopathy, IHD and heart failure continued to be the most seen co-morbidities at 5 years post diagnosis with CKD3a. The remainder of the clinical co-morbidities were not commonly seen five years post diagnosis with CKD3a regardless of the degree of albuminuria (Table 1). Among patients with CKD3aA1, CKD3aA2 and CKD3aA3, 43/224 (19.2%), 30/153 (19.4%) and 26/93 (28%) respectively developed both hypertension and IHD during the 5-year follow-up period from 2016–2021, with no statistically significant difference noted when comparing CKD3aA1 Vs CKD3aA2 (*p* = 0.92), CKD3aA1 Vs CKD3aA3 (*p* = 0.085) and CKD3aA2 Vs CKD3aA3 (*p* = 0.13) (Fig 1).

#### Comparison of co-morbidity at study baseline and endpoint

Statistical analysis revealed there to be a statistically significant difference in the degree of hypertension, diabetic retinopathy, IHD and vascular disease from baseline compared to study end point for all 3 study groups. This was also true for heart failure in patients with CKD3aA1 and CKD3aA3 but not for CKD3aA2 (Table 3).

### Comparison of co-morbidity at study endpoint

Comparing co-morbidity development at study end point amongst study groups, highlighted a statistical difference between CKD3aA1 Vs CKD3aA3 for diabetic retinopathy. Similarly, a statistical difference between CKD3aA2 Vs CKD3aA3 was observed for hypertension and heart failure but not for diabetic retinopathy, IHD or vascular disease (Table 3).

### CKD3a progression analysis

**Fig 2:**
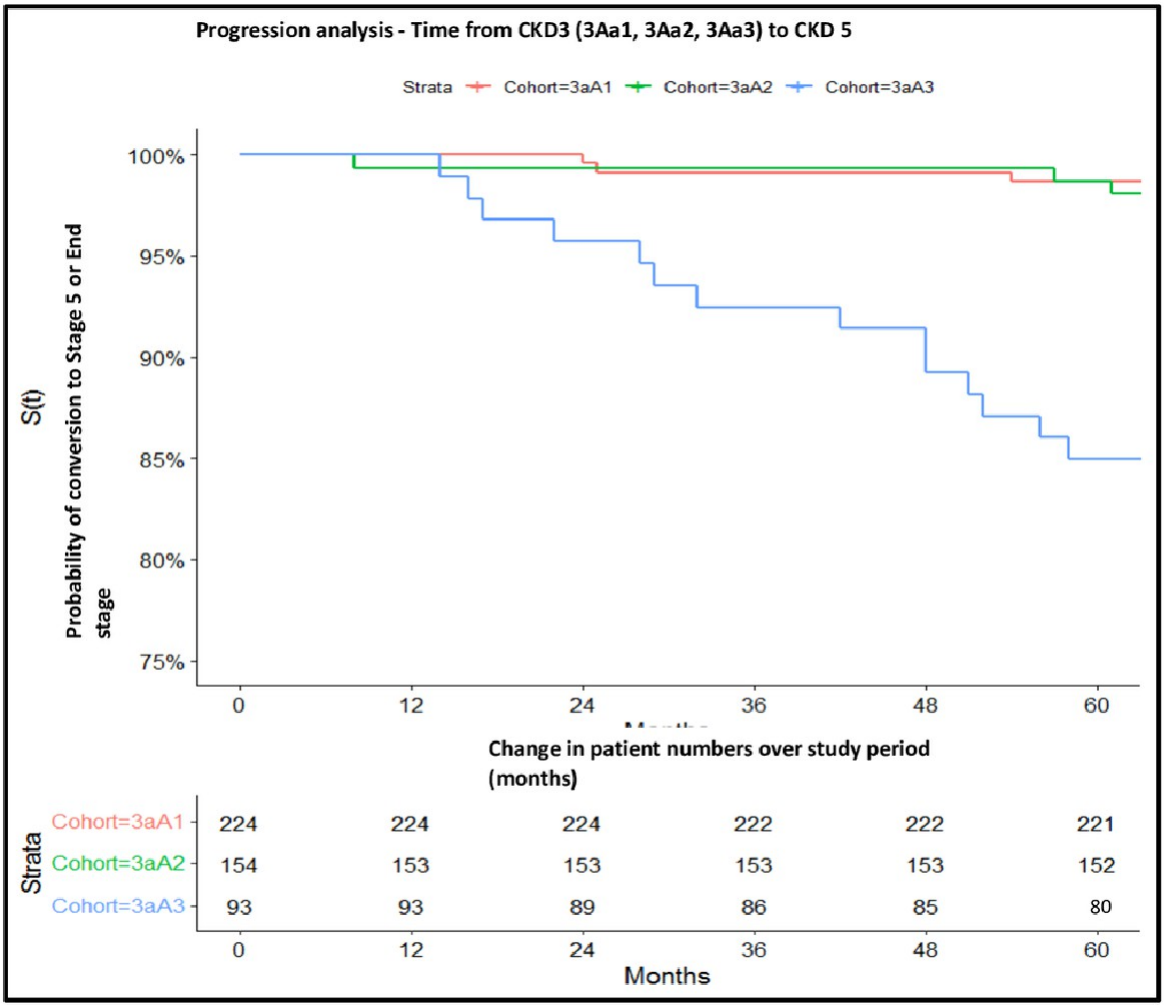
Progression analysis of CKD3aA1/A2/A3 to CKD stage 5 over the 60-month follow-up period between January 2016-December 2021. CKD3aA1 = eGFR between 45-59mls/min/1.73m^2^ with uACR <3mg/mmol, CKD3aA2 = eGFR between 45-59mls/min/1.73m^2^ with uACR between 3-30mg/mmol and CKD3aA3 = eGFR between 45-59mls/min/1.73m^2^ with uACR >30mg/mmol.

Progression analysis of each study cohort showed CKD3aA1 and CKD3aA2 to be associated with a very low degree of progression to CKD 4 or CKD5 over the 5 years follow up period. 3/221 (1.34%), 4/154 (2.6%) and 14/93 (15.1%) of patients with CKD3aA1/2/3 respectively progressed to CKD4. Of those progressing to CKD 4, 3/3(CKD3aA1), 2/4 (CKD3aA2) and 13/14(CKD3aA3) progressed to CKD 5 by 31^st^ December 2021. Thus, progression to CKD5 was most commonly associated with CKD3aA3, with 13/93 (14%) patients progressing to CKD stage 5 within the initial 5 years of diagnosis, perhaps signifying a separate cluster of patients within the CKD3aA3 cohort associated with higher risk of progression to ESKD. (Fig 2) A statistically significant difference was noted when comparing CKD3aA1 Vs CKD3aA3 *p* = <0.00001 and CKD3aA2 Vs CKD3aA3 *p* = <0.00001. No statistically difference was noted when comparing CKD3aA1 VsCKD3aA2 *p* = 0.671427.

### Renal replacement therapy

Amongst the 93 patients with CKD3aA3 in 2015, 13/13 patients reaching CKD 5 required dialysis of which 6 also underwent renal transplantation. No patients were pre-emptively transplanted. Three patients with CKD3aA1 and two patients with CKD3aA2 required renal replacement therapy during the 5 years follow up.

### Prognostic medication utilisation

129/224 (57.6%), 89/154 (57.8%), 60/93 (64.5%) patients with CKD3aA1, CKD3aA2, CKD3aA3 respectively were prescribed a RAASi for a minimum of 3 months in the 12 months prior to the start of the study period between 1/1/2014-31/12/2014. Overall, the utilisation of RAASi and SGLT2i either alone or together in the last six months of the study period between 1/6/2021-31/12/2021 was low, with 113/254(50.4%), 62/154 (40.2%) and 41/93 (44.1%) of patients with CKD3aA1/A2A3 respectively prescribed RAASi and SGLT2i either alone or together Table 4.

**Table 4:** Prescription of RAASi and SGLT2i either singly or together in the last six months of the study period between 1/6/2021-31/12/2021. CKD3aA1 = eGFR between 45-59mls/min/1.73m^2^ with uACR <3mg/mmol, CKD3aA2 = eGFR between 45-59mls/min/1.73m^2^ with uACR between 3-30mg/mmol and CKD3aA3 = eGFR between 45-59mls/min/1.73m^2^ with uACR >30mg/mmol. RAASi = Renin Angiotensin Aldosterone inhibitors. SGLT2i = Sodium-Glucose Cotransporter-2 Inhibitor.

	Prescription for a minimum of 3 months in 2014	Prescription in the last 6 month of study period June-December 2021				
Cohort	RAASi	RAASi	SGLT2i	SGLT2i+RAASi	Total RAASi	Total SGLT2i
3aA1	57.6%	38.4%	2.2%	9.8%	48.2%	12.1%
3aA2	57.8%	30.5%	3.2%	6.5%	37.0%	9.7%
3aA3	64.5%	31.2%	5.4%	7.5%	38.7%	14.0%

CKD3aA1 = eGFR between 45-59mls/min/1.73m^2^ with uACR <3mg/mmol.

CKD3aA2 = eGFR between 45-59mls/min/1.73m^2^ with uACR between 3-30mg/mmol.

CKD3aA3 = eGFR between 45-59mls/min/1.73m^2^ with uACR >30mg/mmol.

RAASi = Renin Angiotensin Aldosterone inhibitor.

SGLT2i = Sodium-Glucose Cotransporter-2 inhibitor.

## Discussion

Our study highlights the multimorbidity associated with CKD3aA1/A2/A3 secondary to DKD at baseline and 5 years post diagnosis. Whilst a significant number of patients were identified to have hypertension, diabetic retinopathy, and IHD at the time of diagnosis with CKD3a, some patients were also identified to have one or more of the other co-morbidities of interest (heart failure, vascular disease, neuropathy, diabetic foot, cataract, liver disease, cerebrovascular disease and amputation), highlighting the high degree of morbidity seen in patients, from the time of initial diagnosis with CKD3a, highlighting the need for early identification of CKD. Whilst all 3 study groups carried a high degree of multimorbidity 5 years post diagnosis, CKD3aA3 was most strongly associated with progression to CKD 5 and more strongly associated with heart failure and hypertension compared to CKD3aA1/A2, highlighting the need for accurate classification of CKD stage by glomerular function and albuminuria, at the time of initial diagnosis to allow for appropriate risk stratification and management.

Whilst the national average in England for uACR testing in T2DM patients between 2015–2016 was reported at 66.7%, in our study cohort 471/1,264 (43.5%) patients with a new eGFR between 45-59ml/min/1.73m(2) underwent assessment for uACR in the same period [11]. This is in keeping with the National CKD audit, where 86% of patients with T2DM underwent an annual blood test, whilst only 54% underwent uACR testing in primary care [9]. With 56.5% of T2DM patients with newly diagnosed CKD3a in 2015, not undergoing uACR testing in the same calendar year as diagnosis with CKD3a by glomerular function, further highlights the need to encourage uACR testing, particularly in primary care, where the vast majority of new CKD diagnoses are made.

The Quality Outcome Framework (QOF) aims to financially incentivise primary care practices to perform particular clinical activities to ensure a good level of care for their patients. However, specifically for CKD, incentives are associated with the coding of CKD stage 3a to 5 based on glomerular function alone. Whilst uACR testing is recommended for patients with CKD and T2DM, it is not associated with a point incentive on the QOF, highlighting a possible reason for suboptimal testing for albuminuria, despite its recommendation [12]. Equally, the relatively asymptomatic course associated with varying degrees of albuminuria alongside a general lack of awareness amongst patients about the importance of the uACR for prognostication may further lead to a lack of uACR being performed by patients, despite it being requested by their community health care team. Thus, new incentives are needed to ensure both primary care health care professionals and patients alike are better supported to ensure testing for both glomerular function and albuminuria in at risk patient groups and subsequent accurate coding on patient’s healthcare records.

The early use of prognostically beneficial medication such as RAASi and SGLT2i in patients with T2DM has the greatest potential to reduce the development of both DKD and CVD. RAASi have since the start of the millennium been instrumental in the management of albuminuric CKD, with trials showing their ability to delay CKD progression [13–15]. Furthermore, their use is associated with a lower rate of major adverse cardiovascular events as well as mortality in patients with CKD and chronic heart failure [16–18]. It is therefore recommended for patients with DKD and a uACR>3mg/mmol, to be initiated on RAASi therapy. However, despite this recommendation, many eligible patients do not receive RAASi therapy, as highlighted by this study where only 37.7% of patients with CKD3aA2/A3 in 2015 were prescribed RAASi therapy between June-December 2021.

Barriers to the prescribing of RAASi in primary care have long been recognised. Clinician concerns regarding hyperkalaemia and an acute reduction in GFR, alongside the requirement for repeated blood tests to ensure glomerular function stability following each dose change in a stretched primary care system with limited capacity, have often been seen to be potential reasons for either the lack of RAASi prescribing or dose optimisation [19]. With RAASi seen to result in a reduction in intraglomerular pressure and thus the degree of albuminuria, and a resultant long term prognostic benefit, significantly more work is needed to be done to ensure all albuminuric diabetic patients are prescribed the maximally tolerated dose of RAASi (as per licence).

The use of SGLT2i is recommended by the National Institute of Clinical Excellence (NICE) in all patients with T2DM deemed to be at increased cardiovascular risk (defined as a QRISK2 of greater than 10%) and in all patients with established chronic heart failure or atherosclerotic heart disease [20]. Similarly following the CREDENCE and DAPA-CKD trials, SGLT2i’s are regularly recommended by nephrologists (according to licence) to reduce progressive renal disease in line with NICE guidance [21, 22]. However despite this, the uptake of SGLT2i prescribing in primary care has been low, with prescribing rates in patients with and without a history of cardiovascular disease ranging between 10–20%, highlighting a delayed shift in primary care practice [23]. Similarly the prescribing of SGLT2i in our cohort has been low, with only 11% patients prescribed SGLT2i in the last 6 months of the study period between June-December 2021. This is however likely secondary to SGLT2i prescribing only recommended by NICE for the first time in November 2021. As a result, the true prescribing practice of SGLT2i in our cohort will be assessed in our follow up study. However, given the inadequate prescribing of RAASi in DKD as demonstrated in our study, it is essential to ensure a rapid uptake of SGLT2i prescribing in primary care, to allow for patients to be able to benefit from their early prescribing and thus reduce overall risk of CKD progression and CVD development. It is therefore essential to increase primary and secondary care awareness and confidence in prescribing SGLT2i (according to licence) to reduce the multimorbidity and mortality associated with DKD.

This study is limited by the relatively short follow up period of five years, with this likely resulting in the lower degree of cerebrovascular disease, vascular disease, diabetic foot disease and amputation than might be expected; a follow-up study is planned to correct for this. Furthermore, the study lacks a true control group of patients with T2DM who do not develop CKD to determine the difference in morbidity associated with T2DM alone Vs T2DM and CKD3aA1/A2andA3. Similarly, a comparison of morbidity outcomes from patients coded with CKD (as done in this study) Vs patients with results indicative of CKD3a but not coded for CKD, would allow for an evaluation of the impact coding in primary care has on patient outcomes. We will thus include this in our planned 10 year follow up study covering the years between 2016 and 2026. Interestingly 57.6% of patients identified to have CKD3aA1 between January-December 2015 had RAASi prescribed for a minimum of 3 months in the 12 months prior to the start of the study period between 1/1/2014-31/12/2014. With RAASi known to reduce the degree of albuminuria, this would have likely reduced the degree of albuminuria identified in those patients prescribed RAASi and thus altered the group into which patients were grouped in this study (i.e. CKD3aA1, CKD3a2, CKD3aA3). This may explain the high degree of heterogeneity and baseline morbidity seen within the CKD3aA1 cohort. This is a limitation of our study and in a subsequent study, where we will longitudinally follow the study cohort over a ten-year period from January 2016 to December 2026, we aim to perform a subgroup analysis of patients who were not prescribed RAASi for a minimum of 3 months in the 12 months prior to the start of the study period.

In summary in this longitudinal study of a multi-ethnic population in NWL, we have shown the ability of regional real-world data in being capable of improving our understanding of multimorbidity development and its management in patients with DKD. The high degree of concurrent hypertension, IHD and retinopathy at the time of diagnosis with CKD3aA1/A2/A3 in our study showcases the importance of primary and secondary prevention in patients with T2DM. Whilst the greatest degree of morbidity at five years post CKD3a diagnosis is associated with CKD3aA3, the morbidity associated with CKD3aA1/A2 should not be underestimated. Ensuring assessment of both uACR and eGFR in those at risk of CKD including patients with T2DM, will allow for patients to be appropriately stratified according to future risk as well as ensuring early diagnosis and management with prognostically beneficial medications, thus delaying and preventing progression of associated morbidity and mortality.

## Supporting information

S1 File T2DM patient characteristics. (DOCX)

S2 File Diagnostic codes. (DOCX)

## References

[1] Chen TK, Knicely DH, Grams ME. Chronic Kidney Disease Diagnosis and Management: A Review. JAMA. 2019 Oct 1;322(13):1294–1304. doi: 10.1001/jama.2019.14745 ; PMCID: PMC7015670. 31573641 PMC7015670

[2] Centers for Disease Control and Prevention: Chronic Kidney Disease in the United States, 2021, Atlanta, GA, US Department of Health and Human Services, Centres for Disease Control and Prevention, 2021

[3] Neuen BL, Weldegiorgis M, Herrington WG, Ohkuma T, Smith M, Woodward M. Changes in GFR and Albuminuria in Routine Clinical Practice and the Risk of Kidney Disease Progression. Am J Kidney Dis. 2021 Sep;78(3):350–360.e1. doi: 10.1053/j.ajkd.2021.02.335. Epub 2021 Apr 23. . 33895181

[4] International Diabetes Federation: Diabetes around the world in 2021. Available at: https://diabetesatlas.org/. Accessed December 12, 2022

[5] uttle KR, Wong L, St Peter W, Roberts G, Rangaswami J, Mottl A, et al.: Moving from evidence to implementation of breakthrough thera-pies for diabetic kidney disease.Clin J Am Soc Nephrol17: 1092–1103,202210.2215/cjn.02980322 10.2215/CJN.02980322 PMC9269635 35649722

[6] Saeedi P, Petersohn I, Salpea P, Malanda B, Karuranga S, Unwin N, et al; IDF Diabetes Atlas Committee. Global and regional diabetes prevalence estimates for 2019 and projections for 2030 and 2045: Results from the International Diabetes Federation Diabetes Atlas, 9^th^edition. Diabetes Res Clin Pract. 2019 Nov;157:107843. doi: 10.1016/j.diabres.2019.107843. Epub 2019 Sep 10. . 31518657

[7] Kidney Disease: Improving Global Outcomes (KDIGO) Diabetes Work Group. KDIGO 2022 Clinical Practice Guideline for Diabetes Management in Chronic Kidney Disease. Kidney Int. 2022;102(5S):S1–S127. 36272764 10.1016/j.kint.2022.06.008

[8] National Institute for Health and Care Excellence. (2021). Chronic kidney disease: assessment and management. [NICE Guideline No. NG203]. https://www.nice.org.uk/guidance/ng203 35077091

[9] Nitsch D, Caplin B, Hull S, Wheeler DC; National CKD Audit and Quality Improvement Programme in Primary Care (2017) First National CKD Audit Report. Available at: https://bit.ly/33hPZVG. Accessed 1th January 2023.

[10] Calibri Light (Headings)Bottle A, Cohen C, Lucas A, et al. How an electronic health record became a real-world research resource: comparison between London’s Whole Systems Integrated Care database and the Clinical Practice Research Datalink. BMC Med Inform Decis Mak. 2020;20(1):71. 10.1186/s12911-020-1082-7 PMC7171852 32312259

[11] National Diabetes Audit, 2015–2016. Report 1: Care Processes and Treatment Targets Available at: https://digital.nhs.uk/data-and-information/publications/statistical/national-diabetes-audit/national-diabetes-audit-2015-2016-report-1-care-processes-and-treatment-targets. Accessed 13th February 2023.

[12] NHS England. Quality and Outcomes Framework guidance for 2021/22. Available at: https://www.england.nhs.uk/wp-content/uploads/2021/03/B0456-update-on-quality-outcomes-framework-changes-for-21-22-.pdf. Accessed 22nd December 2022

[13] Devonald MA, Karet FE. Targeting the renin-angiotensin system in patients with renal disease. J R Soc Med. 2002 Aug;95(8):391–7. doi: 10.1177/014107680209500804 ; PMCID: PMC1279963. 12151488 PMC1279963

[14] Jafar TH, Schmid CH, Landa M, et al.Angiotensin-converting enzyme inhibitors and progression of nondiabetic renal disease. Ann Intern Med 2001;135: 73–87 11453706 10.7326/0003-4819-135-2-200107170-00007

[15] Lewis EJ, Hunsicker LG, Clarke WR, et al.Renoprotective effect of the angiotensin-receptor antagonist irbesartan in patients with nephropathy due to type 2 diabetes. N Engl J Med 2001;345: 851–60 doi: 10.1056/NEJMoa011303 11565517

[16] Heart Outcomes Prevention Evaluation (HOPE) Study Investigators. Effects of ramipril on cardiovascular and microvascular outcomes in people with diabetes mellitus: results of the HOPE study and MICROHOPE substudy. Lancet2000;355: 253–9 10675071

[17] Heart Outcomes Prevention Evaluation Study Investigators, Yusuf S, Sleight P, Pogue J, Bosch J, Davies R, Dagenais G. Effects of an angiotensin-converting-enzyme inhibitor, ramipril, on cardiovascular events in high-risk patients. N Engl J Med. 2000 Jan 20;342(3):145–53. doi: 10.1056/NEJM200001203420301. Erratum in: 2000 May 4;342(18):1376. Erratum in: N Engl J Med 2000 Mar 9;342(10):748. . 10639539

[18] Bosch J, Yusuf S, Pogue J, Sleight P, Lonn E, Rangoonwala B, et al; HOPE Investigators. Heart outcomes prevention evaluation. Use of ramipril in preventing stroke: double blind randomised trial. BMJ. 2002 Mar 23;324(7339):699–702. doi: 10.1136/bmj.324.7339.699 ; PMCID: PMC99052. 11909785 PMC99052

[19] Yildirim T, Arici M, Piskinpasa S, Aybal-Kutlugun A, Yilmaz R, Altun B, et al. Major barriers against renin-angiotensin-aldosterone system blocker use in chronic kidney disease stages 3–5 in clinical practice: a safety concern? Ren Fail. 2012;34(9):1095–9. doi: 10.3109/0886022X.2012.717478. Epub 2012 Sep 6. . 22950572

[20] National Institute for Health and Care Excellence. Type 2 diabetes in adults: management. Published June 2022. Available at: https://www.nice.org.uk/guidance/ng28. (accessed 22/12/22).

[21] Perkovic V, Jardine MJ, Neal B, Bompoint S, Heerspink HJL, Charytan DM, et al; CREDENCE Trial Investigators. Canagliflozin and Renal Outcomes in Type 2 Diabetes and Nephropathy. N Engl J Med. 2019 Jun 13;380(24):2295–2306. doi: 10.1056/NEJMoa1811744. Epub 2019 Apr 14. . 30990260

[22] Heerspink HJL, Stefánsson BV, Correa-Rotter R, Chertow GM, Greene T, Hou FF, et al; DAPA-CKD Trial Committees and Investigators. Dapagliflozin in Patients with Chronic Kidney Disease. N Engl J Med. 2020 Oct 8;383(15):1436–1446. doi: 10.1056/NEJMoa2024816. Epub 2020 Sep 24. . 32970396

[23] Rikin S., Deccy S., Zhang C. et al. Care Gaps in Sodium-Glucose Cotransporter-2 Inhibitor and Renin Angiotensin System Inhibitor Prescriptions for Patients with Diabetic Kidney Disease. J GEN INTERN MED (2022). 10.1007/s11606-022-07863-0 36352203 PMC10212863

