# Correction: Association and progression of multi-morbidity with Chronic Kidney Disease stage 3a secondary to Type 2 Diabetes Mellitus, grouped by albuminuria status in the multi-ethnic population of Northwest London: A real-world study

**DOI:** 10.1371/journal.pone.0341903

**Published:** 2026-01-27

**Authors:** Rakesh Dattani, Zia Ul-Haq, Moulesh Shah, Gabrielle Goldet, Lord Ara Darzi, Hutan Ashrafian, Tahereh Kamalati, Andrew H. Frankel, Frederick W. K. Tam

The Competing Interests statement for this article is incorrect. The correct Competing Interests statement is as follows: RD is supported by Imperial College Institute of Global Health Innovation, Clinical Research Fellowship. FWKT is supported by the Ken and Mary Minton Chair of Renal Medicine. This project is supported by funding from NIH Imperial Biomedical Research Centre and Imperial Health Charity (Diamond fund) and award to FWKT. https://imperialbrc.nihr.ac.uk; https://www.imperialcharity.org.uk.The sponsors did not play a role in the study design, data collection and analysis, decision to publish, or preparation of the manuscript.

## References

[1] Dattani R, Ul-Haq Z, Shah M, Goldet G, Darzi LA, Ashrafian H, et al. Association and progression of multi-morbidity with chronic kidney disease stage 3a secondary to Type 2 Diabetes Mellitus, grouped by albuminuria status in the multi-ethnic population of Northwest London: a real-world study. PLoS One. 2023;18(8):e0289838. doi: 10.1371/journal.pone.0289838 37624842 PMC10456138

