# Supplementary material for: Association and progression of multi-morbidity with Chronic Kidney Disease stage 3a secondary to Type 2 Diabetes Mellitus, grouped by albuminuria status in the multi-ethnic population of Northwest London: A real-world study
Source: PLoS One. 2023 Aug 25;18(8):e0289838. doi: 10.1371/journal.pone.0289838 (PMC10456138; doi:10.1371/journal.pone.0289838)
Supplement: S1 File — (DOCX) [file pone.0289838.s001.docx]

**S1 File - T2DM Patient Characteristics**
