# Supplementary material for: Association and progression of multi-morbidity with Chronic Kidney Disease stage 3a secondary to Type 2 Diabetes Mellitus, grouped by albuminuria status in the multi-ethnic population of Northwest London: A real-world study
Source: PLoS One. 2023 Aug 25;18(8):e0289838. doi: 10.1371/journal.pone.0289838 (PMC10456138; doi:10.1371/journal.pone.0289838)
Supplement: S2 File — (DOCX) [file pone.0289838.s002.docx]

**S2 File - Diagnostic Codes.**

| **CODE** | **Classification** | **Group** |
| --- | --- | --- |
| 7L06. | Read V2 | Amputation |
| 7L062 | Read V2 | Amputation |
| 7L06y | Read V2 | Amputation |
| 7L06z | Read V2 | Amputation |
| 7L07 | Read V2 | Amputation |
| 7L07y | Read V2 | Amputation |
| 7L07z | Read V2 | Amputation |
| 7L080 | Read V2 | Amputation |
| 7L081 | Read V2 | Amputation |
| 7l973 | Read V2 | Amputation |
| 7Lo8y | Read V2 | Amputation |
| X093 | OPCS | Amputation |
| X094 | OPCS | Amputation |
| X095 | OPCS | Amputation |
| X098 | OPCS | Amputation |
| X099 | OPCS | Amputation |
| X101 | OPCS | Amputation |
| X102 | OPCS | Amputation |
| X103 | OPCS | Amputation |
| X104 | OPCS | Amputation |
| X108 | OPCS | Amputation |
| X109 | OPCS | Amputation |
| X111 | OPCS | Amputation |
| X112 | OPCS | Amputation |
| X118 | OPCS | Amputation |
| X119 | OPCS | Amputation |
| X607j | Read V2 | Amputation |
| X6081 | Read V2 | Amputation |
| X6082 | Read V2 | Amputation |
| X6083 | Read V2 | Amputation |
| Xa7Gd | Read V2 | Amputation |
| Xa7GU | Read V2 | Amputation |
| Xa7Gz | Read V2 | Amputation |
| XaBLc | Read V2 | Amputation |
| XaBLT | Read V2 | Amputation |
| XaBLU | Read V2 | Amputation |
| XaBLV | Read V2 | Amputation |
| XaBLW | Read V2 | Amputation |
| XaBLX | Read V2 | Amputation |
| XaBLy | Read V2 | Amputation |
| XaBYC | Read V2 | Amputation |
| XaKK7 | Read V2 | Amputation |
| XE0J7 | Read V2 | Amputation |
| xe0jd | Read V2 | Amputation |
| XE0JE | Read V2 | Amputation |
| XE0JF | Read V2 | Amputation |
| Z894 | ICD10 | Amputation |
| Z894 | ICD10 | Amputation |
| z895 | ICD10 | Amputation |
| z896 | ICD10 | Amputation |
| Z896 | ICD10 | Amputation |
| C108F | Read V2 | cataract |
| C108F | Read V2 | cataract |
| C109E | Read V2 | cataract |
| C109E | Read V2 | cataract |
| C109E | Read V2 | cataract |
| C10EF | Read V2 | cataract |
| C10EF | Read V2 | cataract |
| C10FE | Read V2 | cataract |
| C10FE | Read V2 | cataract |
| G61.. | Read V2 | Cerebrovascular diseases - haemorrhagic |
| G610. | Read V2 | Cerebrovascular diseases - haemorrhagic |
| G611. | Read V2 | Cerebrovascular diseases - haemorrhagic |
| G612. | Read V2 | Cerebrovascular diseases - haemorrhagic |
| G613. | Read V2 | Cerebrovascular diseases - haemorrhagic |
| G614. | Read V2 | Cerebrovascular diseases - haemorrhagic |
| G615. | Read V2 | Cerebrovascular diseases - haemorrhagic |
| G616. | Read V2 | Cerebrovascular diseases - haemorrhagic |
| G618. | Read V2 | Cerebrovascular diseases - haemorrhagic |
| G619. | Read V2 | Cerebrovascular diseases - haemorrhagic |
| G61X. | Read V2 | Cerebrovascular diseases - haemorrhagic |
| G61X0 | Read V2 | Cerebrovascular diseases - haemorrhagic |
| G61X1 | Read V2 | Cerebrovascular diseases - haemorrhagic |
| G61z. | Read V2 | Cerebrovascular diseases - haemorrhagic |
| Gyu62 | Read V2 | Cerebrovascular diseases - haemorrhagic |
| Gyu6F | Read V2 | Cerebrovascular diseases - haemorrhagic |
| I600 | ICD10 | Cerebrovascular diseases - haemorrhagic |
| I601 | ICD10 | Cerebrovascular diseases - haemorrhagic |
| I602 | ICD10 | Cerebrovascular diseases - haemorrhagic |
| I603 | ICD10 | Cerebrovascular diseases - haemorrhagic |
| I604 | ICD10 | Cerebrovascular diseases - haemorrhagic |
| I605 | ICD10 | Cerebrovascular diseases - haemorrhagic |
| I607 | ICD10 | Cerebrovascular diseases - haemorrhagic |
| I608 | ICD10 | Cerebrovascular diseases - haemorrhagic |
| I609 | ICD10 | Cerebrovascular diseases - haemorrhagic |
| I610 | ICD10 | Cerebrovascular diseases - haemorrhagic |
| I611 | ICD10 | Cerebrovascular diseases - haemorrhagic |
| I612 | ICD10 | Cerebrovascular diseases - haemorrhagic |
| I613 | ICD10 | Cerebrovascular diseases - haemorrhagic |
| I614 | ICD10 | Cerebrovascular diseases - haemorrhagic |
| I615 | ICD10 | Cerebrovascular diseases - haemorrhagic |
| I616 | ICD10 | Cerebrovascular diseases - haemorrhagic |
| I618 | ICD10 | Cerebrovascular diseases - haemorrhagic |
| I619 | ICD10 | Cerebrovascular diseases - haemorrhagic |
| I620 | ICD10 | Cerebrovascular diseases - haemorrhagic |
| I621 | ICD10 | Cerebrovascular diseases - haemorrhagic |
| I629 | ICD10 | Cerebrovascular diseases - haemorrhagic |
| I690 | ICD10 | Cerebrovascular diseases - haemorrhagic |
| I691 | ICD10 | Cerebrovascular diseases - haemorrhagic |
| I692 | ICD10 | Cerebrovascular diseases - haemorrhagic |
| Fyu55 | Read V2 | Cerebrovascular diseases - ischaemic |
| G63y0 | Read V2 | Cerebrovascular diseases - ischaemic |
| G63y1 | Read V2 | Cerebrovascular diseases - ischaemic |
| G64.. | Read V2 | Cerebrovascular diseases - ischaemic |
| G640. | Read V2 | Cerebrovascular diseases - ischaemic |
| G6400 | Read V2 | Cerebrovascular diseases - ischaemic |
| G641. | Read V2 | Cerebrovascular diseases - ischaemic |
| G6410 | Read V2 | Cerebrovascular diseases - ischaemic |
| G64z. | Read V2 | Cerebrovascular diseases - ischaemic |
| G64z0 | Read V2 | Cerebrovascular diseases - ischaemic |
| G64z1 | Read V2 | Cerebrovascular diseases - ischaemic |
| G64z2 | Read V2 | Cerebrovascular diseases - ischaemic |
| G64z3 | Read V2 | Cerebrovascular diseases - ischaemic |
| G64z4 | Read V2 | Cerebrovascular diseases - ischaemic |
| G65.. | Read V2 | Cerebrovascular diseases - ischaemic |
| G650. | Read V2 | Cerebrovascular diseases - ischaemic |
| G651. | Read V2 | Cerebrovascular diseases - ischaemic |
| G6510 | Read V2 | Cerebrovascular diseases - ischaemic |
| G652. | Read V2 | Cerebrovascular diseases - ischaemic |
| G653. | Read V2 | Cerebrovascular diseases - ischaemic |
| G654. | Read V2 | Cerebrovascular diseases - ischaemic |
| G656. | Read V2 | Cerebrovascular diseases - ischaemic |
| G657. | Read V2 | Cerebrovascular diseases - ischaemic |
| G65y. | Read V2 | Cerebrovascular diseases - ischaemic |
| G65z. | Read V2 | Cerebrovascular diseases - ischaemic |
| G65zz | Read V2 | Cerebrovascular diseases - ischaemic |
| G660. | Read V2 | Cerebrovascular diseases - ischaemic |
| G661. | Read V2 | Cerebrovascular diseases - ischaemic |
| G662. | Read V2 | Cerebrovascular diseases - ischaemic |
| G663. | Read V2 | Cerebrovascular diseases - ischaemic |
| G664. | Read V2 | Cerebrovascular diseases - ischaemic |
| G665. | Read V2 | Cerebrovascular diseases - ischaemic |
| G666. | Read V2 | Cerebrovascular diseases - ischaemic |
| G6760 | Read V2 | Cerebrovascular diseases - ischaemic |
| G6W.. | Read V2 | Cerebrovascular diseases - ischaemic |
| G6X.. | Read V2 | Cerebrovascular diseases - ischaemic |
| Gyu63 | Read V2 | Cerebrovascular diseases - ischaemic |
| Gyu64 | Read V2 | Cerebrovascular diseases - ischaemic |
| Gyu65 | Read V2 | Cerebrovascular diseases - ischaemic |
| Gyu66 | Read V2 | Cerebrovascular diseases - ischaemic |
| Gyu6G | Read V2 | Cerebrovascular diseases - ischaemic |
| I630 | ICD10 | Cerebrovascular diseases - Ischaemic |
| I631 | ICD10 | Cerebrovascular diseases - Ischaemic |
| I632 | ICD10 | Cerebrovascular diseases - Ischaemic |
| I633 | ICD10 | Cerebrovascular diseases - Ischaemic |
| I634 | ICD10 | Cerebrovascular diseases - Ischaemic |
| I635 | ICD10 | Cerebrovascular diseases - Ischaemic |
| I636 | ICD10 | Cerebrovascular diseases - Ischaemic |
| I638 | ICD10 | Cerebrovascular diseases - Ischaemic |
| I639 | ICD10 | Cerebrovascular diseases - Ischaemic |
| I650 | ICD10 | Cerebrovascular diseases - Ischaemic |
| I651 | ICD10 | Cerebrovascular diseases - Ischaemic |
| I652 | ICD10 | Cerebrovascular diseases - Ischaemic |
| I653 | ICD10 | Cerebrovascular diseases - Ischaemic |
| I658 | ICD10 | Cerebrovascular diseases - Ischaemic |
| I659 | ICD10 | Cerebrovascular diseases - Ischaemic |
| I660 | ICD10 | Cerebrovascular diseases - Ischaemic |
| I661 | ICD10 | Cerebrovascular diseases - Ischaemic |
| I662 | ICD10 | Cerebrovascular diseases - Ischaemic |
| I663 | ICD10 | Cerebrovascular diseases - Ischaemic |
| I664 | ICD10 | Cerebrovascular diseases - Ischaemic |
| I668 | ICD10 | Cerebrovascular diseases - Ischaemic |
| I669 | ICD10 | Cerebrovascular diseases - Ischaemic |
| I693 | ICD10 | Cerebrovascular diseases - Ischaemic |
| ZV12D | Read V2 | Cerebrovascular diseases - ischaemic |
| Fy856 | Read V2 | Cerebrovascular diseases - unspecified |
| G6… | Read V2 | Cerebrovascular diseases - unspecified |
| G62.. | Read V2 | Cerebrovascular diseases - unspecified |
| G66.. | Read V2 | Cerebrovascular diseases - unspecified |
| G667. | Read V2 | Cerebrovascular diseases - unspecified |
| G668. | Read V2 | Cerebrovascular diseases - unspecified |
| G67.. | Read V2 | Cerebrovascular diseases - unspecified |
| G67y. | Read V2 | Cerebrovascular diseases - unspecified |
| I610 | ICD10 | Cerebrovascular diseases - unspecified |
| I611 | ICD10 | Cerebrovascular diseases - unspecified |
| I612 | ICD10 | Cerebrovascular diseases - unspecified |
| I613 | ICD10 | Cerebrovascular diseases - unspecified |
| I614 | ICD10 | Cerebrovascular diseases - unspecified |
| I615 | ICD10 | Cerebrovascular diseases - unspecified |
| I616 | ICD10 | Cerebrovascular diseases - unspecified |
| I618 | ICD10 | Cerebrovascular diseases - unspecified |
| I619 | ICD10 | Cerebrovascular diseases - unspecified |
| I64x | ICD10 | Cerebrovascular diseases - unspecified |
| I64x | ICD10 | Cerebrovascular diseases - unspecified |
| I670 | ICD10 | Cerebrovascular diseases - unspecified |
| I678 | ICD10 | Cerebrovascular diseases - unspecified |
| I694 | ICD10 | Cerebrovascular diseases - unspecified |
| o994 | ICD10 | Cerebrovascular diseases - unspecified |
| X00d1 | Read V2 | Cerebrovascular diseases - unspecified |
| xaEGq | Read V2 | Cerebrovascular diseases - unspecified |
| XE2aB | Read V2 | Cerebrovascular diseases - unspecified |
| z867 | ICD10 | Cerebrovascular diseases - unspecified |
| 1Z10. | Read V2 V2 | Chronic Kidney disease |
| 1Z11. | Read V2 V2 | Chronic Kidney disease |
| 1Z12. | Read V2 V2 | Chronic Kidney disease |
| 1Z13. | Read V2 V2 | Chronic Kidney disease |
| 1Z14. | Read V2 V2 | Chronic Kidney disease |
| K050. | Read V2 V2 | Chronic Kidney disease |
| K051. | Read V2 V2 | Chronic Kidney disease |
| K052. | Read V2 V2 | Chronic Kidney disease |
| K053. | Read V2 V2 | Chronic Kidney disease |
| K054. | Read V2 V2 | Chronic Kidney disease |
| K055. | Read V2 V2 | Chronic Kidney disease |
| K0D.. | Read V2 V2 | Chronic Kidney disease |
| N181 | ICD10 | Chronic Kidney disease |
| N182 | ICD10 | Chronic Kidney disease |
| N183 | ICD10 | Chronic Kidney disease |
| N184 | ICD10 | Chronic Kidney disease |
| N185 | ICD10 | Chronic Kidney disease |
| N186 | ICD10 | Chronic Kidney disease |
| N189 | ICD10 | Chronic Kidney disease |
| C1040 | Read V2 V2 | Diabetic CKD |
| C1041 | Read V2 V2 | Diabetic CKD |
| C104y | Read V2 V2 | Diabetic CKD |
| C1080 | Read V2 V2 | Diabetic CKD |
| C108D | Read V2 V2 | Diabetic CKD |
| C1090 | Read V2 V2 | Diabetic CKD |
| C109C | Read V2 V2 | Diabetic CKD |
| C10E0 | Read V2 V2 | Diabetic CKD |
| C10ED | Read V2 V2 | Diabetic CKD |
| C10EK | Read V2 V2 | Diabetic CKD |
| C10EL | Read V2 V2 | Diabetic CKD |
| C10F0 | Read V2 V2 | Diabetic CKD |
| C10FC | Read V2 V2 | Diabetic CKD |
| C10FL | Read V2 V2 | Diabetic CKD |
| C10FM | Read V2 V2 | Diabetic CKD |
| E112 | ICD10 | Diabetic CKD |
| 2G4E. | Read V2 | diabetic foot |
| 2G510 | Read V2 | diabetic foot |
| 2G510 | Read V2 | diabetic foot |
| 2G510 | Read V2 | diabetic foot |
| 2G5C. | Read V2 | diabetic foot |
| 2G5C. | Read V2 | diabetic foot |
| 2G5C. | Read V2 | diabetic foot |
| 2G5H. | Read V2 | diabetic foot |
| 2G5H. | Read V2 | diabetic foot |
| 2G5H. | Read V2 | diabetic foot |
| 2G5L. | Read V2 | diabetic foot |
| 2G5L. | Read V2 | diabetic foot |
| 2G5L. | Read V2 | diabetic foot |
| 2G5V. | Read V2 | diabetic foot |
| 2G5V. | Read V2 | diabetic foot |
| 2G5W | Read V2 | diabetic foot |
| 2G5W. | Read V2 | diabetic foot |
| 2G5W. | Read V2 | diabetic foot |
| E105 | ICD10 | diabetic foot |
| E105 | ICD10 | diabetic foot |
| E105 | ICD10 | diabetic foot |
| e115 | ICD10 | diabetic foot |
| E115 | ICD10 | diabetic foot |
| E115 | ICD10 | diabetic foot |
| e115 | ICD10 | diabetic foot |
| E115 | ICD10 | diabetic foot |
| e125 | ICD10 | diabetic foot |
| E125 | ICD10 | diabetic foot |
| E125 | ICD10 | diabetic foot |
| e135 | ICD10 | diabetic foot |
| E135 | ICD10 | diabetic foot |
| E135 | ICD10 | diabetic foot |
| e145 | ICD10 | diabetic foot |
| E145 | ICD10 | diabetic foot |
| E145 | ICD10 | diabetic foot |
| L97x | ICD10 | diabetic foot |
| l97x | ICD10 | diabetic foot |
| l97x | ICD10 | diabetic foot |
| l97x | ICD10 | diabetic foot |
| l97x | ICD10 | diabetic foot |
| M0372 | Read V2 | diabetic foot |
| M2710 | Read V2 | diabetic foot |
| M2711 | Read V2 | diabetic foot |
| M2712 | Read V2 | diabetic foot |
| M2714 | Read V2 | diabetic foot |
| M2717 | Read V2 | diabetic foot |
| o241 | ICD10 | diabetic foot |
| o241 | ICD10 | diabetic foot |
| o243 | ICD10 | diabetic foot |
| o243 | ICD10 | diabetic foot |
| o243 | ICD10 | diabetic foot |
| o244 | ICD10 | diabetic foot |
| o244 | ICD10 | diabetic foot |
| o244 | ICD10 | diabetic foot |
| o249 | ICD10 | diabetic foot |
| o249 | ICD10 | diabetic foot |
| o249 | ICD10 | diabetic foot |
| R0542 | Read V2 | diabetic foot |
| R0543 | Read V2 | diabetic foot |
| Z872 | ICD10 | diabetic foot |
| C10B. | Read V2 V2 | Drug Induced Diabetes |
| C10B0 | Read V2 V2 | Drug Induced Diabetes |
| C10H. | Read V2 V2 | Drug Induced Diabetes |
| C10H0 | Read V2 V2 | Drug Induced Diabetes |
| E096 | ICD10 | Drug Induced Diabetes |
| E099 | ICD10 | Drug Induced Diabetes |
| E112 | ICD10 | Drug Induced Diabetes |
| E130 | ICD10 | Drug Induced Diabetes |
| E131 | ICD10 | Drug Induced Diabetes |
| E132 | ICD10 | Drug Induced Diabetes |
| E133 | ICD10 | Drug Induced Diabetes |
| E134 | ICD10 | Drug Induced Diabetes |
| E135 | ICD10 | Drug Induced Diabetes |
| E136 | ICD10 | Drug Induced Diabetes |
| E137 | ICD10 | Drug Induced Diabetes |
| E138 | ICD10 | Drug Induced Diabetes |
| E139 | ICD10 | Drug Induced Diabetes |
| R10C. | Read V2 V2 | Drug Induced Diabetes |
| 2BBF. | Read V2 | eye complication |
| 2BBk. | Read V2 | eye complication |
| 2BBL. | Read V2 | eye complication |
| 2BBl. | Read V2 | eye complication |
| 2BBM. | Read V2 | eye complication |
| 2BBo. | Read V2 | eye complication |
| 2BBP. | Read V2 | eye complication |
| 2BBQ. | Read V2 | eye complication |
| 2BBr. | Read V2 | eye complication |
| 2BBR. | Read V2 | eye complication |
| 2BBS. | Read V2 | eye complication |
| 2BBT. | Read V2 | eye complication |
| 2BBV. | Read V2 | eye complication |
| 2BBW. | Read V2 | eye complication |
| 2BBX. | Read V2 | eye complication |
| C1087 | Read V2 | eye complication |
| C1087 | Read V2 | eye complication |
| C1087 | Read V2 | eye complication |
| C1096 | Read V2 | eye complication |
| C1096 | Read V2 | eye complication |
| C1096 | Read V2 | eye complication |
| C10E7 | Read V2 | eye complication |
| C10E7 | Read V2 | eye complication |
| C10E7 | Read V2 | eye complication |
| C10EP | Read V2 | eye complication |
| C10EP | Read V2 | eye complication |
| C10F0 | Read V2 | eye complication |
| C10F6 | Read V2 | eye complication |
| C10F6 | Read V2 | eye complication |
| C10FQ | Read V2 | eye complication |
| E113 | ICD10 | eye complication |
| 42c.. | Read V2 | HbA1C |
| 42c3. | Read V2 | HbA1C |
| 42W.. | Read V2 | HbA1C |
| 42W.. | Read V2 | HbA1C |
| 42W4. | Read V2 | HbA1C |
| 44TB. | Read V2 | HbA1C |
| 44TC. | Read V2 | HbA1C |
| 44TL. | Read V2 | HbA1C |
| L2859 | Read V2 | HbA1C |
| 14A6. | Read V2 | Heart failure |
| 14AM. | Read V2 | Heart failure |
| 1O1.. | Read V2 | Heart failure |
| 33BA. | Read V2 | Heart failure |
| 388D. | Read V2 | Heart failure |
| 585f. | Read V2 | Heart failure |
| 585g. | Read V2 | Heart failure |
| 661M5 | Read V2 | Heart failure |
| 662F. | Read V2 | Heart Failure |
| 662G. | Read V2 | Heart Failure |
| 662H. | Read V2 | Heart Failure |
| 662I. | Read V2 | Heart Failure |
| 662p. | Read V2 | Heart failure |
| 662T. | Read V2 | Heart failure |
| 662W. | Read V2 | Heart failure |
| 679W1 | Read V2 | Heart failure |
| 8CL3. | Read V2 | Heart failure |
| 8CMK. | Read V2 | Heart failure |
| 8CMW8 | Read V2 | Heart failure |
| 8H2S. | Read V2 | Heart failure |
| 8HBE. | Read V2 | Heart failure |
| 9On.. | Read V2 | Heart failure |
| 9On0. | Read V2 | Heart failure |
| 9On1. | Read V2 | Heart failure |
| 9On2. | Read V2 | Heart failure |
| 9On3. | Read V2 | Heart failure |
| 9On4. | Read V2 | Heart failure |
| 9Or.. | Read V2 | Heart failure |
| 9Or0. | Read V2 | Heart failure |
| 9Or1. | Read V2 | Heart failure |
| 9Or2. | Read V2 | Heart failure |
| 9Or3. | Read V2 | Heart failure |
| 9Or4. | Read V2 | Heart failure |
| 9Or5. | Read V2 | Heart failure |
| ESCTC | Read V2 | Heart failure |
| ESCTC | Read V2 | Heart failure |
| G1yz1 | Read V2 | Heart Failure |
| G2101 | Read V2 | Heart failure |
| G2111 | Read V2 | Heart failure |
| G21z1 | Read V2 | Heart failure |
| G230. | Read V2 | Heart failure |
| G232. | Read V2 | Heart failure |
| G234. | Read V2 | Heart failure |
| G58.. | Read V2 | Heart Failure |
| G580. | Read V2 | Heart Failure |
| G5800 | Read V2 | Heart Failure |
| G5801 | Read V2 | Heart Failure |
| G5802 | Read V2 | Heart Failure |
| G5803 | Read V2 | Heart Failure |
| G5804 | Read V2 | Heart Failure |
| G581. | Read V2 | Heart Failure |
| G5810 | Read V2 | Heart Failure |
| G582. | Read V2 | Heart Failure |
| G583. | Read V2 | Heart Failure |
| G5831 | Read V2 | Heart failure |
| G584. | Read V2 | Heart Failure |
| G58z. | Read V2 | Heart Failure |
| G5yy9 | Read V2 | Heart failure |
| G5yyA | Read V2 | Heart failure |
| G5yyB | Read V2 | Heart failure |
| G5yyC | Read V2 | Heart failure |
| G5yyD | Read V2 | Heart failure |
| G5yyE | Read V2 | Heart failure |
| HNG00 | Read V2 | Heart failure |
| I110 | ICD10 | Heart failure |
| I130 | ICD10 | Heart failure |
| I132 | ICD10 | Heart failure |
| I500 | ICD10 | heart failure |
| I501 | ICD10 | heart failure |
| 6622 | Read V2 | Hypertension |
| 6624 | Read V2 | Hypertension |
| 6627 | Read V2 | Hypertension |
| 6628 | Read V2 | Hypertension |
| 6629 | Read V2 | Hypertension |
| 61462 | Read V2 | Hypertension |
| 14A2. | Read V2 | Hypertension |
| 1JD.. | Read V2 | Hypertension |
| 661M6 | Read V2 | Hypertension |
| 661N6 | Read V2 | Hypertension |
| 662b. | Read V2 | Hypertension |
| 662c. | Read V2 | Hypertension |
| 662d. | Read V2 | Hypertension |
| 662F. | Read V2 | Hypertension |
| 662F. | Read V2 | Hypertension |
| 662G. | Read V2 | Hypertension |
| 662G. | Read V2 | Hypertension |
| 662H. | Read V2 | Hypertension |
| 662O. | Read V2 | Hypertension |
| 662P. | Read V2 | Hypertension |
| 662P0 | Read V2 | Hypertension |
| 662P1 | Read V2 | Hypertension |
| 662q. | Read V2 | Hypertension |
| 662r. | Read V2 | Hypertension |
| 66b2. | Read V2 | Hypertension |
| 67H8. | Read V2 | Hypertension |
| 7Q01. | Read V2 | Hypertension |
| 7Q01y | Read V2 | Hypertension |
| 7Q01z | Read V2 | Hypertension |
| 8B26. | Read V2 | Hypertension |
| 8BL0. | Read V2 | Hypertension |
| 8CR4. | Read V2 | Hypertension |
| 8HT5. | Read V2 | Hypertension |
| 8I3N. | Read V2 | Hypertension |
| 8IA5. | Read V2 | Hypertension |
| 8IA6. | Read V2 | Hypertension |
| 8OAH. | Read V2 | Hypertension |
| 9h3.. | Read V2 | Hypertension |
| 9h31. | Read V2 | Hypertension |
| 9h32. | Read V2 | Hypertension |
| 9N03. | Read V2 | Hypertension |
| 9N1y2 | Read V2 | Hypertension |
| 9N4L. | Read V2 | Hypertension |
| 9OI.. | Read V2 | Hypertension |
| 9OI1. | Read V2 | Hypertension |
| 9OI2. | Read V2 | Hypertension |
| 9OI3. | Read V2 | Hypertension |
| 9OI4. | Read V2 | Hypertension |
| 9OI5. | Read V2 | Hypertension |
| 9OI6. | Read V2 | Hypertension |
| 9OI7. | Read V2 | Hypertension |
| 9OI8. | Read V2 | Hypertension |
| 9OI9. | Read V2 | Hypertension |
| 9OIA. | Read V2 | Hypertension |
| 9OIB. | Read V2 | Hypertension |
| 9OIC. | Read V2 | Hypertension |
| 9OID. | Read V2 | Hypertension |
| 9OIZ. | Read V2 | Hypertension |
| G2... | Read V2 | Hypertension |
| G20.. | Read V2 | Hypertension |
| G200. | Read V2 | Hypertension |
| G201. | Read V2 | Hypertension |
| G202. | Read V2 | Hypertension |
| G203. | Read V2 | Hypertension |
| G20z. | Read V2 | Hypertension |
| G21.. | Read V2 | Hypertension |
| G210. | Read V2 | Hypertension |
| G2100 | Read V2 | Hypertension |
| G2101 | Read V2 | Hypertension |
| G210z | Read V2 | Hypertension |
| G211. | Read V2 | Hypertension |
| G2110 | Read V2 | Hypertension |
| G2111 | Read V2 | Hypertension |
| G211z | Read V2 | Hypertension |
| G21z. | Read V2 | Hypertension |
| G21z0 | Read V2 | Hypertension |
| G21z1 | Read V2 | Hypertension |
| G21zz | Read V2 | Hypertension |
| G22.. | Read V2 | Hypertension |
| G220. | Read V2 | Hypertension |
| G221. | Read V2 | Hypertension |
| G222. | Read V2 | Hypertension |
| G22z. | Read V2 | Hypertension |
| G23.. | Read V2 | Hypertension |
| G230. | Read V2 | Hypertension |
| G231. | Read V2 | Hypertension |
| G232. | Read V2 | Hypertension |
| G233. | Read V2 | Hypertension |
| G234. | Read V2 | Hypertension |
| G23z. | Read V2 | Hypertension |
| G24.. | Read V2 | Hypertension |
| G240. | Read V2 | Hypertension |
| G2400 | Read V2 | Hypertension |
| G240z | Read V2 | Hypertension |
| G241. | Read V2 | Hypertension |
| G2410 | Read V2 | Hypertension |
| G241z | Read V2 | Hypertension |
| G244. | Read V2 | Hypertension |
| G24z. | Read V2 | Hypertension |
| G24z0 | Read V2 | Hypertension |
| G24z1 | Read V2 | Hypertension |
| G24zz | Read V2 | Hypertension |
| G25.. | Read V2 | Hypertension |
| G250. | Read V2 | Hypertension |
| G251. | Read V2 | Hypertension |
| G26.. | Read V2 | Hypertension |
| G261. | Read V2 | Hypertension |
| G27.. | Read V2 | Hypertension |
| G28.. | Read V2 | Hypertension |
| G2y.. | Read V2 | Hypertension |
| G2z.. | Read V2 | Hypertension |
| G672. | Read V2 | Hypertension |
| Gyu2. | Read V2 | Hypertension |
| Gyu20 | Read V2 | Hypertension |
| Gyu21 | Read V2 | Hypertension |
| H4640 | Read V2 | Hypertension |
| H4641 | Read V2 | Hypertension |
| H5832 | Read V2 | Hypertension |
| HNGZ0 | Read V2 | Hypertension |
| I119 | ICD10 | Hypertension |
| I120 | ICD10 | Hypertension |
| I129 | ICD10 | Hypertension |
| I131 | ICD10 | Hypertension |
| I139 | ICD10 | Hypertension |
| I150 | ICD10 | Hypertension |
| I152 | ICD10 | Hypertension |
| I158 | ICD10 | Hypertension |
| I159 | ICD10 | Hypertension |
| L12.. | Read V2 | Hypertension |
| L120. | Read V2 | Hypertension |
| L1200 | Read V2 | Hypertension |
| L1201 | Read V2 | Hypertension |
| L1203 | Read V2 | Hypertension |
| L1204 | Read V2 | Hypertension |
| L120z | Read V2 | Hypertension |
| L121. | Read V2 | Hypertension |
| L1210 | Read V2 | Hypertension |
| L1211 | Read V2 | Hypertension |
| L1212 | Read V2 | Hypertension |
| L1213 | Read V2 | Hypertension |
| L1214 | Read V2 | Hypertension |
| L121z | Read V2 | Hypertension |
| L122. | Read V2 | Hypertension |
| L1220 | Read V2 | Hypertension |
| L1221 | Read V2 | Hypertension |
| L1223 | Read V2 | Hypertension |
| L1224 | Read V2 | Hypertension |
| L122z | Read V2 | Hypertension |
| L123. | Read V2 | Hypertension |
| L1230 | Read V2 | Hypertension |
| L1231 | Read V2 | Hypertension |
| L1232 | Read V2 | Hypertension |
| L1233 | Read V2 | Hypertension |
| L1234 | Read V2 | Hypertension |
| L1235 | Read V2 | Hypertension |
| L1236 | Read V2 | Hypertension |
| L123z | Read V2 | Hypertension |
| L127. | Read V2 | Hypertension |
| L1270 | Read V2 | Hypertension |
| L1271 | Read V2 | Hypertension |
| L1272 | Read V2 | Hypertension |
| L1273 | Read V2 | Hypertension |
| L1274 | Read V2 | Hypertension |
| L127z | Read V2 | Hypertension |
| L128. | Read V2 | Hypertension |
| L1280 | Read V2 | Hypertension |
| L1281 | Read V2 | Hypertension |
| L1282 | Read V2 | Hypertension |
| L12B. | Read V2 | Hypertension |
| L12z. | Read V2 | Hypertension |
| L12z0 | Read V2 | Hypertension |
| L12z1 | Read V2 | Hypertension |
| L12z2 | Read V2 | Hypertension |
| L12z3 | Read V2 | Hypertension |
| L12z4 | Read V2 | Hypertension |
| L12zz | Read V2 | Hypertension |
| Lyu1. | Read V2 | Hypertension |
| Q000. | Read V2 | Hypertension |
| SLC6. | Read V2 | Hypertension |
| SLC6z | Read V2 | Hypertension |
| SyuFT | Read V2 | Hypertension |
| TJC7. | Read V2 | Hypertension |
| TJC7z | Read V2 | Hypertension |
| U60C5 | Read V2 | Hypertension |
| E112 | 3 | ICD10 |
| N083 | 3 | ICD10 |
| N181 | 1 | ICD10 |
| N182 | 2 | ICD10 |
| N183 | 3 | ICD10 |
| N184 | 4 | ICD10 |
| N185 | 5 | ICD10 |
| O241 | 3 | ICD10 |
| 3222 | Read V2 | Ischaemic heart disease |
| 3232 | Read V2 | Ischaemic heart disease |
| 3233 | Read V2 | Ischaemic heart disease |
| 3234 | Read V2 | Ischaemic heart disease |
| 3235 | Read V2 | Ischaemic heart disease |
| 3236 | Read V2 | Ischaemic heart disease |
| 5533 | Read V2 | Ischaemic heart disease |
| 5543 | Read V2 | Ischaemic heart disease |
| 7920 | Read V2 | Ischaemic heart disease |
| 7920 | Read V2 | Ischaemic heart disease |
| 7921 | Read V2 | Ischaemic heart disease |
| 7921 | Read V2 | Ischaemic heart disease |
| 7922 | Read V2 | Ischaemic heart disease |
| 7922 | Read V2 | Ischaemic heart disease |
| 7923 | Read V2 | Ischaemic heart disease |
| 7923 | Read V2 | Ischaemic heart disease |
| 7924 | Read V2 | Ischaemic heart disease |
| 7925 | Read V2 | Ischaemic heart disease |
| 7925 | Read V2 | Ischaemic heart disease |
| 7926 | Read V2 | Ischaemic heart disease |
| 7928 | Read V2 | Ischaemic heart disease |
| 7928 | Read V2 | Ischaemic heart disease |
| 7928 | Read V2 | Ischaemic heart disease |
| 79200 | Read V2 | Ischaemic heart disease |
| 79201 | Read V2 | Ischaemic heart disease |
| 79202 | Read V2 | Ischaemic heart disease |
| 79203 | Read V2 | Ischaemic heart disease |
| 79210 | Read V2 | Ischaemic heart disease |
| 79211 | Read V2 | Ischaemic heart disease |
| 79212 | Read V2 | Ischaemic heart disease |
| 79213 | Read V2 | Ischaemic heart disease |
| 79220 | Read V2 | Ischaemic heart disease |
| 79221 | Read V2 | Ischaemic heart disease |
| 79222 | Read V2 | Ischaemic heart disease |
| 79223 | Read V2 | Ischaemic heart disease |
| 79230 | Read V2 | Ischaemic heart disease |
| 79231 | Read V2 | Ischaemic heart disease |
| 79232 | Read V2 | Ischaemic heart disease |
| 79233 | Read V2 | Ischaemic heart disease |
| 79240 | Read V2 | Ischaemic heart disease |
| 79241 | Read V2 | Ischaemic heart disease |
| 79242 | Read V2 | Ischaemic heart disease |
| 79250 | Read V2 | Ischaemic heart disease |
| 79251 | Read V2 | Ischaemic heart disease |
| 79253 | Read V2 | Ischaemic heart disease |
| 79253 | Read V2 | Ischaemic heart disease |
| 79253 | Read V2 | Ischaemic heart disease |
| 79254 | Read V2 | Ischaemic heart disease |
| 79260 | Read V2 | Ischaemic heart disease |
| 79262 | Read V2 | Ischaemic heart disease |
| 79263 | Read V2 | Ischaemic heart disease |
| 79275 | Read V2 | Ischaemic heart disease |
| 79280 | Read V2 | Ischaemic heart disease |
| 79280 | Read V2 | Ischaemic heart disease |
| 79281 | Read V2 | Ischaemic heart disease |
| 79281 | Read V2 | Ischaemic heart disease |
| 79282 | Read V2 | Ischaemic heart disease |
| 79282 | Read V2 | Ischaemic heart disease |
| 79283 | Read V2 | Ischaemic heart disease |
| 79290 | Read V2 | Ischaemic heart disease |
| 79290 | Read V2 | Ischaemic heart disease |
| 79291 | Read V2 | Ischaemic heart disease |
| 79291 | Read V2 | Ischaemic heart disease |
| 79291 | Read V2 | Ischaemic heart disease |
| 79293 | Read V2 | Ischaemic heart disease |
| 79293 | Read V2 | Ischaemic heart disease |
| 79294 | Read V2 | Ischaemic heart disease |
| 79294 | Read V2 | Ischaemic heart disease |
| 79295 | Read V2 | Ischaemic heart disease |
| 79295 | Read V2 | Ischaemic heart disease |
| 79296 | Read V2 | Ischaemic heart disease |
| 14A3. | Read V2 | Ischaemic heart disease |
| 14A4. | Read V2 | Ischaemic heart disease |
| 14A5. | Read V2 | Ischaemic heart disease |
| 14AH. | Read V2 | Ischaemic heart disease |
| 14AJ. | Read V2 | Ischaemic heart disease |
| 14AL. | Read V2 | Ischaemic heart disease |
| 14AT. | Read V2 | Ischaemic heart disease |
| 14AW. | Read V2 | Ischaemic heart disease |
| 187.. | Read V2 | Ischaemic heart disease |
| 322.. | Read V2 | Ischaemic heart disease |
| 322Z. | Read V2 | Ischaemic heart disease |
| 323.. | Read V2 | Ischaemic heart disease |
| 323Z. | Read V2 | Ischaemic heart disease |
| 32B.. | Read V2 | Ischaemic heart disease |
| 32B2. | Read V2 | Ischaemic heart disease |
| 32B3. | Read V2 | Ischaemic heart disease |
| 32BZ. | Read V2 | Ischaemic heart disease |
| 661M0 | Read V2 | Ischaemic heart disease |
| 662K. | Read V2 | Ischaemic heart disease |
| 662K0 | Read V2 | Ischaemic heart disease |
| 662K1 | Read V2 | Ischaemic heart disease |
| 662K2 | Read V2 | Ischaemic heart disease |
| 662K3 | Read V2 | Ischaemic heart disease |
| 662Kz | Read V2 | Ischaemic heart disease |
| 662N. | Read V2 | Ischaemic heart disease |
| 6A2.. | Read V2 | Ischaemic heart disease |
| 6A4.. | Read V2 | Ischaemic heart disease |
| 792.. | Read V2 | Ischaemic heart disease |
| 7920y | Read V2 | Ischaemic heart disease |
| 7920z | Read V2 | Ischaemic heart disease |
| 7921y | Read V2 | Ischaemic heart disease |
| 7921z | Read V2 | Ischaemic heart disease |
| 7922y | Read V2 | Ischaemic heart disease |
| 7922z | Read V2 | Ischaemic heart disease |
| 7923z | Read V2 | Ischaemic heart disease |
| 7924y | Read V2 | Ischaemic heart disease |
| 7924z | Read V2 | Ischaemic heart disease |
| 7925y | Read V2 | Ischaemic heart disease |
| 7925z | Read V2 | Ischaemic heart disease |
| 7926z | Read V2 | Ischaemic heart disease |
| 7928y | Read V2 | Ischaemic heart disease |
| 7928y | Read V2 | Ischaemic heart disease |
| 7928z | Read V2 | Ischaemic heart disease |
| 7928z | Read V2 | Ischaemic heart disease |
| 7929y | Read V2 | Ischaemic heart disease |
| 7929z | Read V2 | Ischaemic heart disease |
| 792B0 | Read V2 | Ischaemic heart disease |
| 792C. | Read V2 | Ischaemic heart disease |
| 792C0 | Read V2 | Ischaemic heart disease |
| 792Cy | Read V2 | Ischaemic heart disease |
| 792Cz | Read V2 | Ischaemic heart disease |
| 792D. | Read V2 | Ischaemic heart disease |
| 792Dy | Read V2 | Ischaemic heart disease |
| 792Dz | Read V2 | Ischaemic heart disease |
| 793G. | Read V2 | Ischaemic heart disease |
| 793G. | Read V2 | Ischaemic heart disease |
| 793G0 | Read V2 | Ischaemic heart disease |
| 793G0 | Read V2 | Ischaemic heart disease |
| 793G1 | Read V2 | Ischaemic heart disease |
| 793G1 | Read V2 | Ischaemic heart disease |
| 793G2 | Read V2 | Ischaemic heart disease |
| 793G2 | Read V2 | Ischaemic heart disease |
| 793G3 | Read V2 | Ischaemic heart disease |
| 793G3 | Read V2 | Ischaemic heart disease |
| 793Gy | Read V2 | Ischaemic heart disease |
| 793Gy | Read V2 | Ischaemic heart disease |
| 793Gz | Read V2 | Ischaemic heart disease |
| 793Gz | Read V2 | Ischaemic heart disease |
| 7A4B8 | Read V2 | Ischaemic heart disease |
| 7A540 | Read V2 | Ischaemic heart disease |
| 7A545 | Read V2 | Ischaemic heart disease |
| 8B3k. | Read V2 | Ischaemic heart disease |
| 8CMP. | Read V2 | Ischaemic heart disease |
| 8H2V. | Read V2 | Ischaemic heart disease |
| 8I37. | Read V2 | Ischaemic heart disease |
| 8IEY. | Read V2 | Ischaemic heart disease |
| 8L40. | Read V2 | Ischaemic heart disease |
| 8L41. | Read V2 | Ischaemic heart disease |
| 8T04. | Read V2 | Ischaemic heart disease |
| 9Ob.. | Read V2 | Ischaemic heart disease |
| 9Ob0. | Read V2 | Ischaemic heart disease |
| 9Ob1. | Read V2 | Ischaemic heart disease |
| 9Ob2. | Read V2 | Ischaemic heart disease |
| 9Ob3. | Read V2 | Ischaemic heart disease |
| 9Ob4. | Read V2 | Ischaemic heart disease |
| 9Ob5. | Read V2 | Ischaemic heart disease |
| 9Ob6. | Read V2 | Ischaemic heart disease |
| 9Ob8. | Read V2 | Ischaemic heart disease |
| 9Ob9. | Read V2 | Ischaemic heart disease |
| A360 | ICD10 | Ischaemic heart disease |
| A691 | ICD10 | Ischaemic heart disease |
| B085 | ICD10 | Ischaemic heart disease |
| B270 | ICD10 | Ischaemic heart disease |
| B271 | ICD10 | Ischaemic heart disease |
| B278 | ICD10 | Ischaemic heart disease |
| B279 | ICD10 | Ischaemic heart disease |
| D70x | ICD10 | Ischaemic heart disease |
| E116 | ICD10 | Ischaemic heart disease |
| G3... | Read V2 | Ischaemic heart disease |
| G30.. | Read V2 | Ischaemic heart disease |
| G30.. | Read V2 | Ischaemic heart disease |
| G30.. | Read V2 | Ischaemic heart disease |
| G30.. | Read V2 | Ischaemic heart disease |
| G30.. | Read V2 | Ischaemic heart disease |
| G30.. | Read V2 | Ischaemic heart disease |
| G30.. | Read V2 | Ischaemic heart disease |
| G30.. | Read V2 | Ischaemic heart disease |
| G300. | Read V2 | Ischaemic heart disease |
| G301. | Read V2 | Ischaemic heart disease |
| G3010 | Read V2 | Ischaemic heart disease |
| G3011 | Read V2 | Ischaemic heart disease |
| G3011 | Read V2 | Ischaemic heart disease |
| G301z | Read V2 | Ischaemic heart disease |
| G302. | Read V2 | Ischaemic heart disease |
| G303. | Read V2 | Ischaemic heart disease |
| G304. | Read V2 | Ischaemic heart disease |
| G305. | Read V2 | Ischaemic heart disease |
| G306. | Read V2 | Ischaemic heart disease |
| G307. | Read V2 | Ischaemic heart disease |
| G3070 | Read V2 | Ischaemic heart disease |
| G3071 | Read V2 | Ischaemic heart disease |
| G308. | Read V2 | Ischaemic heart disease |
| G309. | Read V2 | Ischaemic heart disease |
| G30B. | Read V2 | Ischaemic heart disease |
| G30X. | Read V2 | Ischaemic heart disease |
| G30X0 | Read V2 | Ischaemic heart disease |
| G30y. | Read V2 | Ischaemic heart disease |
| G30y0 | Read V2 | Ischaemic heart disease |
| G30y1 | Read V2 | Ischaemic heart disease |
| G30y2 | Read V2 | Ischaemic heart disease |
| G30yz | Read V2 | Ischaemic heart disease |
| G30z. | Read V2 | Ischaemic heart disease |
| G31.. | Read V2 | Ischaemic heart disease |
| G311. | Read V2 | Ischaemic heart disease |
| G3110 | Read V2 | Ischaemic heart disease |
| G3111 | Read V2 | Ischaemic heart disease |
| G3112 | Read V2 | Ischaemic heart disease |
| G3113 | Read V2 | Ischaemic heart disease |
| G3114 | Read V2 | Ischaemic heart disease |
| G3115 | Read V2 | Ischaemic heart disease |
| G311z | Read V2 | Ischaemic heart disease |
| G312. | Read V2 | Ischaemic heart disease |
| G31y. | Read V2 | Ischaemic heart disease |
| G31y0 | Read V2 | Ischaemic heart disease |
| G31y1 | Read V2 | Ischaemic heart disease |
| G31y2 | Read V2 | Ischaemic heart disease |
| G31y3 | Read V2 | Ischaemic heart disease |
| G31yz | Read V2 | Ischaemic heart disease |
| G32.. | Read V2 | Ischaemic heart disease |
| G33.. | Read V2 | Ischaemic heart disease |
| G330. | Read V2 | Ischaemic heart disease |
| G3300 | Read V2 | Ischaemic heart disease |
| G330z | Read V2 | Ischaemic heart disease |
| G33z. | Read V2 | Ischaemic heart disease |
| G33z0 | Read V2 | Ischaemic heart disease |
| G33z1 | Read V2 | Ischaemic heart disease |
| G33z2 | Read V2 | Ischaemic heart disease |
| G33z3 | Read V2 | Ischaemic heart disease |
| G33z4 | Read V2 | Ischaemic heart disease |
| G33z5 | Read V2 | Ischaemic heart disease |
| G33z6 | Read V2 | Ischaemic heart disease |
| G33z7 | Read V2 | Ischaemic heart disease |
| G33zz | Read V2 | Ischaemic heart disease |
| G34.. | Read V2 | Ischaemic heart disease |
| G340. | Read V2 | Ischaemic heart disease |
| G340. | Read V2 | Ischaemic heart disease |
| G340. | Read V2 | Ischaemic heart disease |
| G3400 | Read V2 | Ischaemic heart disease |
| G3401 | Read V2 | Ischaemic heart disease |
| G342. | Read V2 | Ischaemic heart disease |
| G343. | Read V2 | Ischaemic heart disease |
| G344. | Read V2 | Ischaemic heart disease |
| G34y. | Read V2 | Ischaemic heart disease |
| G34y0 | Read V2 | Ischaemic heart disease |
| G34y1 | Read V2 | Ischaemic heart disease |
| G34yz | Read V2 | Ischaemic heart disease |
| G34z. | Read V2 | Ischaemic heart disease |
| G34z0 | Read V2 | Ischaemic heart disease |
| G35.. | Read V2 | Ischaemic heart disease |
| G350. | Read V2 | Ischaemic heart disease |
| G351. | Read V2 | Ischaemic heart disease |
| G353. | Read V2 | Ischaemic heart disease |
| G35X. | Read V2 | Ischaemic heart disease |
| G38.. | Read V2 | Ischaemic heart disease |
| G380. | Read V2 | Ischaemic heart disease |
| G381. | Read V2 | Ischaemic heart disease |
| G382. | Read V2 | Ischaemic heart disease |
| G383. | Read V2 | Ischaemic heart disease |
| G384. | Read V2 | Ischaemic heart disease |
| G38z. | Read V2 | Ischaemic heart disease |
| G39.. | Read V2 | Ischaemic heart disease |
| G3y.. | Read V2 | Ischaemic heart disease |
| G3z.. | Read V2 | Ischaemic heart disease |
| Gyu3. | Read V2 | Ischaemic heart disease |
| Gyu30 | Read V2 | Ischaemic heart disease |
| Gyu32 | Read V2 | Ischaemic heart disease |
| Gyu33 | Read V2 | Ischaemic heart disease |
| Gyu34 | Read V2 | Ischaemic heart disease |
| Gyu36 | Read V2 | Ischaemic heart disease |
| I200 | ICD10 | Ischaemic heart disease |
| I200 | ICD10 | Ischaemic heart disease |
| I200 | ICD10 | Ischaemic heart disease |
| I201 | ICD10 | Ischaemic heart disease |
| I201 | ICD10 | Ischaemic heart disease |
| I201 | ICD10 | Ischaemic heart disease |
| I208 | ICD10 | Ischaemic heart disease |
| I208 | ICD10 | Ischaemic heart disease |
| I208 | ICD10 | Ischaemic heart disease |
| I208 | ICD10 | Ischaemic heart disease |
| I209 | ICD10 | Ischaemic heart disease |
| I209 | ICD10 | Ischaemic heart disease |
| I209 | ICD10 | Ischaemic heart disease |
| I209 | ICD10 | Ischaemic heart disease |
| I210 | ICD10 | Ischaemic heart disease |
| I211 | ICD10 | Ischaemic heart disease |
| I212 | ICD10 | Ischaemic heart disease |
| I213 | ICD10 | Ischaemic heart disease |
| I214 | ICD10 | Ischaemic heart disease |
| I219 | ICD10 | Ischaemic heart disease |
| I220 | ICD10 | Ischaemic heart disease |
| I221 | ICD10 | Ischaemic heart disease |
| I228 | ICD10 | Ischaemic heart disease |
| I229 | ICD10 | Ischaemic heart disease |
| I230 | ICD10 | Ischaemic heart disease |
| I231 | ICD10 | Ischaemic heart disease |
| I232 | ICD10 | Ischaemic heart disease |
| I233 | ICD10 | Ischaemic heart disease |
| I234 | ICD10 | Ischaemic heart disease |
| I235 | ICD10 | Ischaemic heart disease |
| I236 | ICD10 | Ischaemic heart disease |
| I238 | ICD10 | Ischaemic heart disease |
| I240 | ICD10 | Ischaemic heart disease |
| I248 | ICD10 | Ischaemic heart disease |
| I249 | ICD10 | Ischaemic heart disease |
| I250 | ICD10 | Ischaemic heart disease |
| I251 | ICD10 | Ischaemic heart disease |
| I252 | ICD10 | Ischaemic heart disease |
| I254 | ICD10 | Ischaemic heart disease |
| I255 | ICD10 | Ischaemic heart disease |
| I256 | ICD10 | Ischaemic heart disease |
| I258 | ICD10 | Ischaemic heart disease |
| I259 | ICD10 | Ischaemic heart disease |
| I2C2. | Read V2 | Ischaemic heart disease |
| I2c3. | Read V2 | Ischaemic heart disease |
| I739 | ICD10 | Ischaemic heart disease |
| J020 | ICD10 | Ischaemic heart disease |
| J312 | ICD10 | Ischaemic heart disease |
| K122 | ICD10 | Ischaemic heart disease |
| K401 | OPCS | ischaemic heart disease |
| K402 | OPCS | ischaemic heart disease |
| K403 | OPCS | ischaemic heart disease |
| K404 | OPCS | ischaemic heart disease |
| K409 | OPCS | ischaemic heart disease |
| K411 | OPCS | ischaemic heart disease |
| K412 | OPCS | ischaemic heart disease |
| K413 | OPCS | ischaemic heart disease |
| K414 | OPCS | ischaemic heart disease |
| K419 | OPCS | ischaemic heart disease |
| K421 | OPCS | ischaemic heart disease |
| K422 | OPCS | ischaemic heart disease |
| K423 | OPCS | ischaemic heart disease |
| K429 | OPCS | ischaemic heart disease |
| K431 | OPCS | ischaemic heart disease |
| K432 | OPCS | ischaemic heart disease |
| K433 | OPCS | ischaemic heart disease |
| K434 | OPCS | ischaemic heart disease |
| K439 | OPCS | ischaemic heart disease |
| K441 | OPCS | ischaemic heart disease |
| K442 | OPCS | ischaemic heart disease |
| K448 | OPCS | ischaemic heart disease |
| K449 | OPCS | ischaemic heart disease |
| K451 | OPCS | ischaemic heart disease |
| K452 | OPCS | ischaemic heart disease |
| K453 | OPCS | ischaemic heart disease |
| K454 | OPCS | ischaemic heart disease |
| K455 | OPCS | ischaemic heart disease |
| K456 | OPCS | ischaemic heart disease |
| K458 | OPCS | ischaemic heart disease |
| K459 | OPCS | ischaemic heart disease |
| K461 | OPCS | ischaemic heart disease |
| K463 | OPCS | ischaemic heart disease |
| K468 | OPCS | ischaemic heart disease |
| K469 | OPCS | ischaemic heart disease |
| K491 | OPCS | ischaemic heart disease |
| K492 | OPCS | ischaemic heart disease |
| K493 | OPCS | ischaemic heart disease |
| K494 | OPCS | ischaemic heart disease |
| K498 | OPCS | ischaemic heart disease |
| K499 | OPCS | ischaemic heart disease |
| K501 | OPCS | ischaemic heart disease |
| K502 | OPCS | ischaemic heart disease |
| K503 | OPCS | ischaemic heart disease |
| K504 | OPCS | ischaemic heart disease |
| K508 | OPCS | ischaemic heart disease |
| K509 | OPCS | ischaemic heart disease |
| K751 | OPCS | ischaemic heart disease |
| K752 | OPCS | ischaemic heart disease |
| K753 | OPCS | ischaemic heart disease |
| K754 | OPCS | ischaemic heart disease |
| K758 | OPCS | ischaemic heart disease |
| K759 | OPCS | ischaemic heart disease |
| o241 | ICD10 | Ischaemic heart disease |
| SP003 | Read V2 | Ischaemic heart disease |
| SP076 | Read V2 | Ischaemic heart disease |
| T828 | ICD10 | Ischaemic heart disease |
| X2008 | Read V2 | Ischaemic heart disease |
| X200A | Read V2 | Ischaemic heart disease |
| XE0oG | Read V2 | Ischaemic heart disease |
| Z824 | ICD10 | Ischaemic heart disease |
| Z824 | ICD10 | Ischaemic heart disease |
| ZV457 | Read V2 | Ischaemic heart disease |
| ZV458 | Read V2 | Ischaemic heart disease |
| ZV45K | Read V2 | Ischaemic heart disease |
| ZV45K | Read V2 | Ischaemic heart disease |
| ZV45L | Read V2 | Ischaemic heart disease |
| J61y. | Read V2 | Liver |
| J61y1 | Read V2 | Liver |
| J61y7 | Read V2 | Liver |
| J61y8 | Read V2 | Liver |
| J61y9 | Read V2 | Liver |
| J61y9 | Read V2 | Liver |
| J61yz | Read V2 | Liver |
| K758 | ICD10 | Liver |
| K760 | ICD10 | Liver |
| 14o8. | Read V2 | NDH |
| 6AC.. | Read V2 | NDH |
| C11y2 | Read V2 | NDH |
| C11y3 | Read V2 | NDH |
| C11y4 | Read V2 | NDH |
| C11y5 | Read V2 | NDH |
| C317. | Read V2 | NDH |
| o998 | ICD11 | NDH |
| R10C. | Read V2 | NDH |
| R10D0 | Read V2 | NDH |
| R10D1 | Read V2 | NDH |
| R10E. | Read V2 | NDH |
| R730 | ICD10 | NDH |
| 1M8.. | Read V2 | neuropathy |
| C1060 | Read V2 | neuropathy |
| C1061 | Read V2 | neuropathy |
| C1061 | Read V2 | neuropathy |
| C1062 | Read V2 | neuropathy |
| C1069 | Read V2 | neuropathy |
| C106y | Read V2 | neuropathy |
| C106y | Read V2 | neuropathy |
| C106z | Read V2 | neuropathy |
| C1082 | Read V2 | neuropathy |
| C1082 | Read V2 | neuropathy |
| C1082 | Read V2 | neuropathy |
| C108B | Read V2 | neuropathy |
| C108B | Read V2 | neuropathy |
| C108H | Read V2 | neuropathy |
| C108H | Read V2 | neuropathy |
| C108J | Read V2 | neuropathy |
| C108J | Read V2 | neuropathy |
| C108J | Read V2 | neuropathy |
| C1092 | Read V2 | neuropathy |
| C1092 | Read V2 | neuropathy |
| C1092 | Read V2 | neuropathy |
| C1092 | Read V2 | neuropathy |
| C1092 | Read V2 | neuropathy |
| C1092 | Read V2 | neuropathy |
| C109A | Read V2 | neuropathy |
| C109A | Read V2 | neuropathy |
| C109A | Read V2 | neuropathy |
| C109A | Read V2 | neuropathy |
| C109A | Read V2 | neuropathy |
| C109B | Read V2 | neuropathy |
| C109B | Read V2 | neuropathy |
| C109B | Read V2 | neuropathy |
| C109B | Read V2 | neuropathy |
| C109B | Read V2 | neuropathy |
| C109G | Read V2 | neuropathy |
| C109G | Read V2 | neuropathy |
| C109G | Read V2 | neuropathy |
| C109G | Read V2 | neuropathy |
| C109G | Read V2 | neuropathy |
| C109H | Read V2 | neuropathy |
| C109H | Read V2 | neuropathy |
| C109H | Read V2 | neuropathy |
| C109H | Read V2 | neuropathy |
| C10E2 | Read V2 | neuropathy |
| C10E2 | Read V2 | neuropathy |
| C10EB | Read V2 | neuropathy |
| C10EC | Read V2 | neuropathy |
| C10EC | Read V2 | neuropathy |
| C10EC | Read V2 | neuropathy |
| C10EH | Read V2 | neuropathy |
| C10EJ | Read V2 | neuropathy |
| C10EQ | Read V2 | neuropathy |
| C10EQ | Read V2 | neuropathy |
| C10F2 | Read V2 | neuropathy |
| C10F2 | Read V2 | neuropathy |
| C10F2 | Read V2 | neuropathy |
| C10F2 | Read V2 | neuropathy |
| C10FA | Read V2 | neuropathy |
| C10FA | Read V2 | neuropathy |
| C10FA | Read V2 | neuropathy |
| C10FA | Read V2 | neuropathy |
| C10FB | Read V2 | neuropathy |
| C10FB | Read V2 | neuropathy |
| C10FB | Read V2 | neuropathy |
| C10FG | Read V2 | neuropathy |
| C10FG | Read V2 | neuropathy |
| C10FG | Read V2 | neuropathy |
| C10FG | Read V2 | neuropathy |
| C10FH | Read V2 | neuropathy |
| C10FH | Read V2 | neuropathy |
| C10FH | Read V2 | neuropathy |
| C10FR | Read V2 | neuropathy |
| F1711 | Read V2 | neuropathy |
| F372. | Read V2 | neuropathy |
| F3720 | Read V2 | neuropathy |
| F3720 | Read V2 | neuropathy |
| F3721 | Read V2 | neuropathy |
| F3721 | Read V2 | neuropathy |
| F3722 | Read V2 | neuropathy |
| F3722 | Read V2 | neuropathy |
| F3y0. | Read V2 | neuropathy |
| G73y0 | Read V2 | neuropathy |
| C10N1 | Read V2 V2 | Secondary - Cystic fibrosis |
| E840 | ICD10 | Secondary - Cystic fibrosis |
| C10G. | Read V2 V2 | Secondary - Pancreatic |
| C10G0 | Read V2 V2 | Secondary - Pancreatic |
| C259 | ICD10 | Secondary - Pancreatic |
| E831 | ICD10 | Secondary - Pancreatic |
| K859 | ICD10 | Secondary - Pancreatic |
| C10N. | Read V2 V2 | Secondary - Unknown |
| C10N0 | Read V2 V2 | Secondary - Unknown |
| E089 | ICD10 | Secondary - Unknown |
| E220 | ICD10 | Secondary - Unknown |
| L1809 | Read V2 V2 | Secondary - Unknown |
| G606 | Read V2 | Subarachnoid Haemorrhage - SHA |
| G60z. | Read V2 | Subarachnoid Haemorrhage - SHA |
| Gyu60 | Read V2 | Subarachnoid Haemorrhage - SHA |
| Gyu61 | Read V2 | Subarachnoid Haemorrhage - SHA |
| Gyu6E | Read V2 | Subarachnoid Haemorrhage - SHA |
| I600 | ICD10 | Subarachnoid Haemorrhage - SHA |
| I601 | ICD10 | Subarachnoid Haemorrhage - SHA |
| I602 | ICD10 | Subarachnoid Haemorrhage - SHA |
| I603 | ICD10 | Subarachnoid Haemorrhage - SHA |
| I604 | ICD10 | Subarachnoid Haemorrhage - SHA |
| I605 | ICD10 | Subarachnoid Haemorrhage - SHA |
| I607 | ICD10 | Subarachnoid Haemorrhage - SHA |
| I608 | ICD10 | Subarachnoid Haemorrhage - SHA |
| I609 | ICD10 | Subarachnoid Haemorrhage - SHA |
| I690 | ICD10 | Subarachnoid Haemorrhage - SHA |
| X00Dg | Read V2 | Subarachnoid Haemorrhage - SHA |
| Xa01b | Read V2 | Subarachnoid Haemorrhage - SHA |
| Xa01c | Read V2 | Subarachnoid Haemorrhage - SHA |
| Xa01h | Read V2 | Subarachnoid Haemorrhage - SHA |
| Xa01i | Read V2 | Subarachnoid Haemorrhage - SHA |
| Xa01j | Read V2 | Subarachnoid Haemorrhage - SHA |
| Xa01k | Read V2 | Subarachnoid Haemorrhage - SHA |
| Xa01l | Read V2 | Subarachnoid Haemorrhage - SHA |
| Xa01m | Read V2 | Subarachnoid Haemorrhage - SHA |
| Xa01o | Read V2 | Subarachnoid Haemorrhage - SHA |
| Xa0N7 | Read V2 | Subarachnoid Haemorrhage - SHA |
| Xa1uW | Read V2 | Subarachnoid Haemorrhage - SHA |
| XE2bF | Read V2 | Subarachnoid Haemorrhage - SHA |
| C1000 | Read V2 V2 | Type1 Diabetes |
| C108A | Read V2 V2 | Type1 Diabetes |
| C10E. | Read V2 V2 | Type1 Diabetes |
| C10E3 | Read V2 V2 | Type1 Diabetes |
| C10E4 | Read V2 V2 | Type1 Diabetes |
| C10E8 | Read V2 V2 | Type1 Diabetes |
| C10E9 | Read V2 V2 | Type1 Diabetes |
| C10EA | Read V2 V2 | Type1 Diabetes |
| C10EE | Read V2 V2 | Type1 Diabetes |
| C10EM | Read V2 V2 | Type1 Diabetes |
| C10EN | Read V2 V2 | Type1 Diabetes |
| E100 | ICD10 | Type1 Diabetes |
| E101 | ICD10 | Type1 Diabetes |
| E102 | ICD10 | Type1 Diabetes |
| E103 | ICD10 | Type1 Diabetes |
| E104 | ICD10 | Type1 Diabetes |
| E105 | ICD10 | Type1 Diabetes |
| E106 | ICD10 | Type1 Diabetes |
| E107 | ICD10 | Type1 Diabetes |
| E108 | ICD10 | Type1 Diabetes |
| E109 | ICD10 | Type1 Diabetes |
| 66A4. | Read V2 V2 | Type2 Diabetes |
| C1001 | Read V2 V2 | Type2 Diabetes |
| C1021 | Read V2 V2 | Type2 Diabetes |
| C1031 | Read V2 V2 | Type2 Diabetes |
| C1051 | Read V2 V2 | Type2 Diabetes |
| C1061 | Read V2 V2 | Type2 Diabetes |
| C1074 | Read V2 V2 | Type2 Diabetes |
| C1089 | Read V2 V2 | Type2 Diabetes |
| C109. | Read V2 V2 | Type2 Diabetes |
| C1090 | Read V2 V2 | Type2 Diabetes |
| C1091 | Read V2 V2 | Type2 Diabetes |
| C1092 | Read V2 V2 | Type2 Diabetes |
| C1093 | Read V2 V2 | Type2 Diabetes |
| C1094 | Read V2 V2 | Type2 Diabetes |
| C1095 | Read V2 V2 | Type2 Diabetes |
| C1096 | Read V2 V2 | Type2 Diabetes |
| C1097 | Read V2 V2 | Type2 Diabetes |
| C1099 | Read V2 V2 | Type2 Diabetes |
| C109A | Read V2 V2 | Type2 Diabetes |
| C109B | Read V2 V2 | Type2 Diabetes |
| C109C | Read V2 V2 | Type2 Diabetes |
| C109D | Read V2 V2 | Type2 Diabetes |
| C109E | Read V2 V2 | Type2 Diabetes |
| C109F | Read V2 V2 | Type2 Diabetes |
| C109G | Read V2 V2 | Type2 Diabetes |
| C109H | Read V2 V2 | Type2 Diabetes |
| C109J | Read V2 V2 | Type2 Diabetes |
| C109K | Read V2 V2 | Type2 Diabetes |
| C10F. | Read V2 V2 | Type2 Diabetes |
| C10F0 | Read V2 V2 | Type2 Diabetes |
| C10F1 | Read V2 V2 | Type2 Diabetes |
| C10F2 | Read V2 V2 | Type2 Diabetes |
| C10F3 | Read V2 V2 | Type2 Diabetes |
| C10F4 | Read V2 V2 | Type2 Diabetes |
| C10F5 | Read V2 V2 | Type2 Diabetes |
| C10F6 | Read V2 V2 | Type2 Diabetes |
| C10F7 | Read V2 V2 | Type2 Diabetes |
| C10F9 | Read V2 V2 | Type2 Diabetes |
| C10FA | Read V2 V2 | Type2 Diabetes |
| C10FB | Read V2 V2 | Type2 Diabetes |
| C10FC | Read V2 V2 | Type2 Diabetes |
| C10FD | Read V2 V2 | Type2 Diabetes |
| C10FE | Read V2 V2 | Type2 Diabetes |
| C10FF | Read V2 V2 | Type2 Diabetes |
| C10FG | Read V2 V2 | Type2 Diabetes |
| C10FH | Read V2 V2 | Type2 Diabetes |
| C10FJ | Read V2 V2 | Type2 Diabetes |
| C10FK | Read V2 V2 | Type2 Diabetes |
| C10FL | Read V2 V2 | Type2 Diabetes |
| C10FM | Read V2 V2 | Type2 Diabetes |
| C10FN | Read V2 V2 | Type2 Diabetes |
| C10FP | Read V2 V2 | Type2 Diabetes |
| C10FQ | Read V2 V2 | Type2 Diabetes |
| C10FR | Read V2 V2 | Type2 Diabetes |
| E110 | ICD10 | Type2 Diabetes |
| E111 | ICD10 | Type2 Diabetes |
| E113 | ICD10 | Type2 Diabetes |
| E114 | ICD10 | Type2 Diabetes |
| E115 | ICD10 | Type2 Diabetes |
| E116 | ICD10 | Type2 Diabetes |
| E117 | ICD10 | Type2 Diabetes |
| E118 | ICD10 | Type2 Diabetes |
| E119 | ICD10 | Type2 Diabetes |
| L1806 | Read V2 V2 | Type2 Diabetes |
| 1M110 | Read V2 | vascular disease |
| 1M111 | Read V2 | vascular disease |
| 24EA. | Read V2 | vascular disease |
| 24FA. | Read V2 | vascular disease |
| 2G63 | Read V2 | vascular disease |
| 7A100 | Read V2 | vascular disease |
| 7A101 | Read V2 | vascular disease |
| 7A102 | Read V2 | vascular disease |
| 7A103 | Read V2 | vascular disease |
| 7A104 | Read V2 | vascular disease |
| 7A10y | Read V2 | vascular disease |
| 7A10z | Read V2 | vascular disease |
| 7A121 | Read V2 | vascular disease |
| 7A121 | Read V2 | vascular disease |
| 7A123 | Read V2 | vascular disease |
| 7A123 | Read V2 | vascular disease |
| 7A190 | Read V2 | vascular disease |
| 7A191 | Read V2 | vascular disease |
| 7A1A1 | Read V2 | vascular disease |
| 7A41 | Read V2 | vascular disease |
| 7A411 | Read V2 | vascular disease |
| 7A412 | Read V2 | vascular disease |
| 7A413 | Read V2 | vascular disease |
| 7A420 | Read V2 | vascular disease |
| 7A420 | Read V2 | vascular disease |
| 7A421 | Read V2 | vascular disease |
| 7A421 | Read V2 | vascular disease |
| 7A430 | Read V2 | vascular disease |
| 7A431 | Read V2 | vascular disease |
| 7A431 | Read V2 | vascular disease |
| 7A441 | Read V2 | vascular disease |
| 7A471 | Read V2 | vascular disease |
| 7A472 | Read V2 | vascular disease |
| 7A473 | Read V2 | vascular disease |
| 7A474 | Read V2 | vascular disease |
| 7A475 | Read V2 | vascular disease |
| 7A476 | Read V2 | vascular disease |
| 7A481 | Read V2 | vascular disease |
| 7A482 | Read V2 | vascular disease |
| 7A483 | Read V2 | vascular disease |
| 7A484 | Read V2 | vascular disease |
| 7A485 | Read V2 | vascular disease |
| 7A486 | Read V2 | vascular disease |
| 7A491 | Read V2 | vascular disease |
| 7A492 | Read V2 | vascular disease |
| 7A493 | Read V2 | vascular disease |
| 7A494 | Read V2 | vascular disease |
| 7A495 | Read V2 | vascular disease |
| 7A4A1 | Read V2 | vascular disease |
| 7A4A2 | Read V2 | vascular disease |
| 7A4A2 | Read V2 | vascular disease |
| 7A4A3 | Read V2 | vascular disease |
| 7A4A3 | Read V2 | vascular disease |
| 7A4B1 | Read V2 | vascular disease |
| 7A4B2 | Read V2 | vascular disease |
| 7A523 | Read V2 | vascular disease |
| 7A541 | Read V2 | vascular disease |
| C107. | Read V2 | vascular disease |
| C1071 | Read V2 | vascular disease |
| C1071 | Read V2 | vascular disease |
| C1074 | Read V2 | vascular disease |
| C1074 | Read V2 | vascular disease |
| C1079 | Read V2 | vascular disease |
| C107y | Read V2 | vascular disease |
| C107z | Read V2 | vascular disease |
| C108G | Read V2 | vascular disease |
| C109F | Read V2 | vascular disease |
| C109F | Read V2 | vascular disease |
| C109F | Read V2 | vascular disease |
| C109F | Read V2 | vascular disease |
| C109F | Read V2 | vascular disease |
| C10F5 | Read V2 | vascular disease |
| C10FF | Read V2 | vascular disease |
| C10FF | Read V2 | vascular disease |
| C10FF | Read V2 | vascular disease |
| C10FF | Read V2 | vascular disease |
| E115 | ICD10 | vascular disease |
| E125 | ICD10 | vascular disease |
| E135 | ICD10 | vascular disease |
| E145 | ICD10 | vascular disease |
| G7001 | Read V2 | vascular disease |
| G73.. | Read V2 | vascular disease |
| G731. | Read V2 | vascular disease |
| G732. | Read V2 | vascular disease |
| G733. | Read V2 | vascular disease |
| G734. | Read V2 | vascular disease |
| G73y. | Read V2 | vascular disease |
| G73z. | Read V2 | vascular disease |
| G73z0 | Read V2 | vascular disease |
| G73z0 | Read V2 | vascular disease |
| G73zz | Read V2 | vascular disease |
| G7402 | Read V2 | vascular disease |
| Gyu74 | Read V2 | vascular disease |
| HNG00 | Read V2 | vascular disease |
| HNG01 | Read V2 | vascular disease |
| I700 | ICD10 | Vascular Disease |
| I7000 | ICD10 | Vascular Disease |
| I7001 | ICD10 | Vascular Disease |
| I701 | ICD10 | Vascular Disease |
| I7010 | ICD10 | Vascular Disease |
| I7011 | ICD10 | Vascular Disease |
| I702 | ICD10 | Vascular Disease |
| I7020 | ICD10 | Vascular Disease |
| I7021 | ICD10 | Vascular Disease |
| I708 | ICD10 | Vascular Disease |
| I709 | ICD10 | Vascular Disease |
| I7091 | ICD10 | Vascular Disease |
| I710 | ICD10 | Vascular Disease |
| I711 | ICD10 | Vascular Disease |
| I712 | ICD10 | Vascular Disease |
| I713 | ICD10 | Vascular Disease |
| I714 | ICD10 | Vascular Disease |
| I715 | ICD10 | Vascular Disease |
| I716 | ICD10 | Vascular Disease |
| I718 | ICD10 | Vascular Disease |
| I719 | ICD10 | Vascular Disease |
| I720 | ICD10 | Vascular Disease |
| I721 | ICD10 | Vascular Disease |
| I722 | ICD10 | Vascular Disease |
| I723 | ICD10 | Vascular Disease |
| I724 | ICD10 | Vascular Disease |
| I725 | ICD10 | Vascular Disease |
| I726 | ICD10 | Vascular Disease |
| I728 | ICD10 | Vascular Disease |
| I729 | ICD10 | Vascular Disease |
| I738 | ICD10 | Vascular Disease |
| I739 | ICD10 | Vascular Disease |
| I740 | ICD10 | Vascular Disease |
| I741 | ICD10 | Vascular Disease |
| I742 | ICD10 | Vascular Disease |
| I743 | ICD10 | Vascular Disease |
| I744 | ICD10 | Vascular Disease |
| I745 | ICD10 | Vascular Disease |
| I748 | ICD10 | Vascular Disease |
| I749 | ICD10 | Vascular Disease |
| I771 | ICD10 | Vascular Disease |
| I772 | ICD10 | Vascular Disease |
| I778 | ICD10 | Vascular Disease |
| I779 | ICD10 | Vascular Disease |
| I790 | ICD10 | Vascular Disease |
| 1Z10. | Read V2 | CKD 1 |
| 1Z17. | Read V2 | CKD 1 |
| 1Z18. | Read V2 | CKD 1 |
| K051. | Read V2 | CKD 1 |
| 1Z1M. | Read V2 | CKD 1 |
| 1Z1N. | Read V2 | CKD 1 |
| 1Z1P. | Read V2 | CKD 1 |
| 1Z11. | Read V2 | CKD 2 |
| 1Z19. | Read V2 | CKD 2 |
| 1Z1A. | Read V2 | CKD 2 |
| K052. | Read V2 | CKD 2 |
| 1Z1Q. | Read V2 | CKD 2 |
| 1Z1R. | Read V2 | CKD 2 |
| 1Z1S. | Read V2 | CKD 2 |
| 1Z12. | Read V2 | CKD 3 |
| K053. | Read V2 | CKD 3 |
| 1z1C | Read V2 | CKD 3 |
| 1z1B | Read V2 | CKD 3 |
| 1Z15 | Read V2 | CKD 3 |
| 1z16 | Read V2 | CKD 3 |
| 1z1e | Read V2 | CKD 3 |
| 1z1g | Read V2 | CKD 3 |
| 1Z1F | Read V2 | CKD 3 |
| 1z1v | Read V2 | CKD 3 |
| 1z1d | Read V2 | CKD 3 |
| 1z1w | Read V2 | CKD 3 |
| 1z1z | Read V2 | CKD 3 |
| 1z1y | Read V2 | CKD 3 |
| 1z1x | Read V2 | CKD 3 |
| 1z1t | Read V2 | CKD 3 |
| 1Z13. | Read V2 | CKD 4 |
| 1Z1H. | Read V2 | CKD 4 |
| 1Z1J. | Read V2 | CKD 4 |
| K054. | Read V2 | CKD 4 |
| 1Z1a. | Read V2 | CKD 4 |
| 1Z1c | Read V2 | CKD 4 |
| 1Z1b | Read V2 | CKD 4 |
| 1Z14. | Read V2 | CKD 5 |
| 1Z1K. | Read V2 | CKD 5 |
| 1Z1L.00 | Read V2 | CKD 5 |
| K055. | Read V2 | CKD 5 |
| 1Z1e | Read V2 | CKD 5 |
| **K05..12** | Read V2 | CKD 5 |
| **1Z1d.00** | Read V2 | CKD 5 |
| **1Z1f.00** | Read V2 | CKD 5 |
| **K050.00** | Read V2 | CKD 5 |
| 1Z1L.11 | Read V2 | CKD 5 |
| 451K. | Read V2 | eGFR |
| 451M. | Read V2 | eGFR |
| 451N. | Read V2 | eGFR |
|  | Read V2 | eGFR |
| **451N.** | Read V2 | eGFR |
| 451E | Read V2 | eGFR |
| 451G | Read V2 | eGFR |
| 451F | Read V2 | eGFR |
| 451K | Read V2 | eGFR |
| 451M | Read V2 | eGFR |
| 7L1B.11 | Read V2 | Dialysis for renal failure |
| ZV56y11 | Read V2 | Dialysis for renal failure |
| 7L1A000 | Read V2 | Dialysis for renal failure |
| 14V2.11 | Read V2 | Dialysis for renal failure |
| 7L1A400 | Read V2 | Dialysis for renal failure |
| 7L1A200 | Read V2 | Dialysis for renal failure |
| 7L1A.11 | Read V2 | Dialysis for renal failure |
| 7L1f000 | Read V2 | Dialysis for renal failure |
| ZV56.00 | Read V2 | Dialysis for renal failure |
| 7L1C000 | Read V2 | Dialysis for renal failure |
| 7L1B000 | Read V2 | Dialysis for renal failure |
| 7L1A100 | Read V2 | Dialysis for renal failure |
| 7L1A500 | Read V2 | Dialysis for renal failure |
| 14V2.00 | Read V2 | Dialysis for renal failure |
| ZVu3G00 | Read V2 | Dialysis for renal failure |
| 7L1A600 | Read V2 | Dialysis for renal failure |
| 761A0 | Read V2 | Dialysis for renal failure |
| 761A1 | Read V2 | Dialysis for renal failure |
| 761A2 | Read V2 | Dialysis for renal failure |
| 761A4 | Read V2 | Dialysis for renal failure |
| 761A5 | Read V2 | Dialysis for renal failure |
| Y84.1 | Read V2 | Dialysis for renal failure |
| Z49 | Read V2 | Dialysis for renal failure |
| TB11. | Read V2 | Dialysis for renal failure |
| Z49.1 | Read V2 | Dialysis for renal failure |
| Z49.2 | Read V2 | Dialysis for renal failure |
| Z99.2 | Read V2 | Dialysis for renal failure |
| 7L1A1 | Read V2 | Dialysis for renal failure |
| Ua1IM | Read V2 | Dialysis for renal failure |
| Ua1IN | Read V2 | Dialysis for renal failure |
| Z992 | Read V2 | Dialysis for renal failure |
| N185 | Read V2 | Dialysis for renal failure |
| Z491 | Read V2 | CKD 5 |
| E112D | Read V2 | CKD 5 |
| Z491 | Read V2 | CKD 5 |
| N083A | Read V2 | CKD 5 |
| E112D | Read V2 | CKD 5 |
| N083A | Read V2 | CKD 5 |
| 7B001 | Read V2 | Transplant of kidney |
| 7B00. | Read V2 | Transplant of kidney |
| ZV420 | Read V2 | Transplant of kidney |
| 7B00.00 | Read V2 | Transplant of kidney |
| 7B00100 | Read V2 | Transplant of kidney |
| 7B00200 | Read V2 | Transplant of kidney |
| 7B00211 | Read V2 | Transplant of kidney |
| 7B00212 | Read V2 | Transplant of kidney |
| 7B00300 | Read V2 | Transplant of kidney |
| 7B00400 | Read V2 | Transplant of kidney |
| 7B00y | Read V2 | Transplant of kidney |
| 7B00z | Read V2 | Transplant of kidney |
| 7B002 | Read V2 | Transplant of kidney |
| 7B00100 | Read V2 | Transplant of kidney |
| 7B00111 | Read V2 | Transplant of kidney |
| 7B00600 | Read V2 | Transplant of kidney |
| 7B00y00 | Read V2 | Transplant of kidney |
| 7B00z00 | Read V2 | Transplant of kidney |
| 7B0F.00 | Read V2 | Transplant of kidney |
| 7B0F300 | Read V2 | Transplant of kidney |
| 7B0Fy00 | Read V2 | Transplant of kidney |
| 7B0Fz00 | Read V2 | Transplant of kidney |
| TB00100 | Read V2 | Transplant of kidney |
| TB00111 | Read V2 | Transplant of kidney |
| ZV42000 | Read V2 | Transplant of kidney |
| 7B063 | Read V2 | Transplant of kidney |
| 7B015 | Read V2 | Transplant of kidney |
| 7B019 | Read V2 | Transplant of kidney |
| N18.1 | ICD10 | CKD1 |
| N18.2 | ICD10 | CKD2 |
| N18.3 | ICD10 | CKD3 |
| o241 | ICD10 | CKD3 |
| e112d | ICD10 | CKD3 |
| n083a | ICD10 | CKD3 |
| N18.4 | ICD10 | CKD4 |
| N18.5 | ICD10 | CKD5 |
| z940 | ICD10 | Tranplant of kidney |
| N185 | ICD10 | Tranplant of kidney |
| 431855005 | SNOMED | CKD 1 |
| 3.68421E+14 | SNOMED | CKD 1 |
| 1.17681E+14 | SNOMED | CKD 1 |
| 9.0721E+13 | SNOMED | CKD 1 |
| 7.51E+11 | SNOMED | CKD 1 |
| 3.24121E+14 | SNOMED | CKD 1 |
| 3.24151E+14 | SNOMED | CKD 1 |
| 9.49401E+14 | SNOMED | CKD 1 |
| 431856006 | SNOMED | CKD 2 |
| 3.68431E+14 | SNOMED | CKD 2 |
| 1.29181E+14 | SNOMED | CKD 2 |
| 9.0731E+13 | SNOMED | CKD 2 |
| 7.41E+11 | SNOMED | CKD 2 |
| 3.24181E+14 | SNOMED | CKD 2 |
| 3.24211E+14 | SNOMED | CKD 2 |
| 949521000000108 | SNOMED | CKD 2 |
| 949561000000100 | SNOMED | CKD 2 |
| 9.49621E+14 | SNOMED | CKD 2 |
| 433144002 | SNOMED | CKD 3 |
| 6.91421E+14 | SNOMED | CKD 3 |
| 3.68441E+14 | SNOMED | CKD 3 |
| 1.29171E+14 | SNOMED | CKD 3 |
| 9.0741E+13 | SNOMED | CKD 3 |
| 7.31E+11 | SNOMED | CKD 3 |
| 3.24251E+14 | SNOMED | CKD 3 |
| 3.24281E+14 | SNOMED | CKD 3 |
| 700378005 | SNOMED | CKD 3 |
| 700379002 | SNOMED | CKD 3 |
| 3.24371E+14 | SNOMED | CKD 3 |
| 9.50061E+14 | SNOMED | CKD 3 |
|  | SNOMED | CKD 3 |
|  | SNOMED | CKD 3 |
| 3.24341E+14 | SNOMED | CKD 3 |
| 3.24311E+14 | SNOMED | CKD 3 |
| 9.49881E+14 | SNOMED | CKD 3 |
| 3.24411E+14 | SNOMED | CKD 3 |
| 3.24341E+14 | SNOMED | CKD 3 |
| 431857002 | SNOMED | CKD 4 |
| 6.91401E+14 | SNOMED | CKD 4 |
| 3.68451E+14 | SNOMED | CKD 4 |
| 1.29151E+14 | SNOMED | CKD 4 |
| 9.0751E+13 | SNOMED | CKD 4 |
| 7.21E+11 | SNOMED | CKD 4 |
| 3.24441E+14 | SNOMED | CKD 4 |
| 3.24471E+14 | SNOMED | CKD 4 |
| 9.50181E+14 | SNOMED | CKD 4 |
| 433146000 | SNOMED | CKD 5 |
| 3.68461E+14 | SNOMED | CKD 5 |
| 1.29161E+14 | SNOMED | CKD 5 |
| 9.0761E+13 | SNOMED | CKD 5 |
| 7.11E+11 | SNOMED | CKD 5 |
| 714152005 | SNOMED | CKD 5 |
| 714153000 | SNOMED | CKD 5 |
| )950251000000106 | SNOMED | CKD 5 |
| 950291000000103 | SNOMED | CKD 5 |
| 9.50311E+14 | SNOMED | CKD 5 |
| 3.68461E+14 | SNOMED | CKD 5 |
| 1.29161E+14 | SNOMED | CKD 5 |
| 9.0761E+13 | SNOMED | CKD 5 |
| 7.11E+11 | SNOMED | CKD 5 |
| 714152005 | SNOMED | CKD 5 |
| 3.24501E+14 | SNOMED | CKD 5 |
| 714153000 | SNOMED | CKD 5 |
| 3.24541E+14 | SNOMED | CKD 5 |
| 8.57971E+14 | SNOMED | eGFR |
| 9.63601E+14 | SNOMED | eGFR |
| 1.01149E+15 | SNOMED | eGFR |
| 9.63621E+14 | SNOMED | eGFR |
| 1.01148E+15 | SNOMED | eGFR |
